# Classification of radiation effects for dose limitation purposes: history, current situation and future prospects

**DOI:** 10.1093/jrr/rru019

**Published:** 2014-05-03

**Authors:** Nobuyuki Hamada, Yuki Fujimichi

**Affiliations:** Radiation Safety Research Center, Nuclear Technology Research Laboratory, Central Research Institute of Electric Power Industry (CRIEPI), 2-11-1 Iwado-kita, Komae, Tokyo 201-8511, Japan

**Keywords:** tissue reactions, stochastic effects, threshold, detriment, life-threatening effects, non-life-threatening effects

## Abstract

Radiation exposure causes cancer and non-cancer health effects, each of which differs greatly in the shape of the dose–response curve, latency, persistency, recurrence, curability, fatality and impact on quality of life. In recent decades, for dose limitation purposes, the International Commission on Radiological Protection has divided such diverse effects into tissue reactions (formerly termed non-stochastic and deterministic effects) and stochastic effects. On the one hand, effective dose limits aim to reduce the risks of stochastic effects (cancer/heritable effects) and are based on the detriment-adjusted nominal risk coefficients, assuming a linear-non-threshold dose response and a dose and dose rate effectiveness factor of 2. On the other hand, equivalent dose limits aim to avoid tissue reactions (vision-impairing cataracts and cosmetically unacceptable non-cancer skin changes) and are based on a threshold dose. However, the boundary between these two categories is becoming vague. Thus, we review the changes in radiation effect classification, dose limitation concepts, and the definition of detriment and threshold. Then, the current situation is overviewed focusing on (i) stochastic effects with a threshold, (ii) tissue reactions without a threshold, (iii) target organs/tissues for circulatory disease, (iv) dose levels for limitation of cancer risks vs prevention of non-life-threatening tissue reactions vs prevention of life-threatening tissue reactions, (v) mortality or incidence of thyroid cancer, and (vi) the detriment for tissue reactions. For future discussion, one approach is suggested that classifies radiation effects according to whether effects are life threatening, and radiobiological research needs are also briefly discussed.

## INTRODUCTION

Ionizing radiation has long been indispensable in medicine and industry. As soon as Röntgen submitted the first paper announcing his discovery of X-rays in December 1895, human exposure to a large dose of radiation took place without knowledge on its physical quantity and health effects. The first case of human dermatitis of the hand was reported in January 1896 [1], followed by various reports of other harmful effects. To avoid the dangers of overexposure, the International X-ray and Radium Protection Committee (IXRPC) issued the first recommendations in 1928 [2]. Since then, the IXRPC and its successor, the International Commission on Radiological Protection (ICRP) have developed a system of radiological protection (RP). The current recommendations issued as ICRP Publication 103 (ICRP-103) [3] aim to contribute to an appropriate level of protection against the detrimental effects of radiation exposure without unduly limiting the desirable human actions.

Radiation exposure causes cancer and non-cancer health effects, each of which varies greatly in the shape of the dose–response curve, latency, persistency, recurrence, curability, fatality and impact on quality of life (QOL). In recent decades, for dose limitation purposes, ICRP has divided all such diverse radiation effects into either stochastic effects (with no threshold) or tissue reactions (formerly termed non-stochastic or deterministic effects, which do have a threshold). On the one hand, effective dose limits aim at reducing the risks of stochastic effects (cancer/heritable effects) and are based on detriment-adjusted nominal risk coefficients, assuming a linear-non-threshold (LNT) dose response and a dose and dose rate effectiveness factor (DDREF) of 2. On the other hand, equivalent dose limits aim at avoiding tissue reactions [vision-impairing cataract (VIC) and cosmetically unacceptable non-cancer skin changes] and are based on a threshold dose.

In 2011, the ICRP issued the Seoul Statement [4] to lower the threshold for cataracts to 0.5 Gy and the occupational equivalent dose limit for the lens to 20 mSv/year (averaged over defined periods of 5 years with no single year exceeding 50 mSv) and to recommend the first ever threshold of 0.5 Gy for cardiovascular disease (CVD) and cerebrovascular disease (CeVD) to the heart and brain, respectively. These new thresholds for cataracts and fatal circulatory disease came at least in part from epidemiological evidence demonstrating an LNT dose response [4–7], and have also stimulated discussions on the detriment for tissue reactions and target organs/tissues for circulatory disease [8]. Taken together, some human cancers exhibit non-linear dose responses [9, 10]. Such current situation makes the distinction between stochastic effects and tissue reactions fuzzy, necessitating the clarification and discussion of current issues and future needs to improve the situation.

This paper briefly reviews the changes in the classification of radiation effects, concepts for dose limitation, and definition of detriment and threshold, and overviews the current situation we are facing. For discussion, one approach is suggested that classifies radiation effects according to whether or not effects are life threatening. Future radiobiological research needs shall also be discussed.

## CHANGES IN RADIATION EFFECT CLASSIFICATION

In the 1928 Recommendations [2], injuries to the superficial tissues, derangements of internal organs and changes in the blood were listed as ‘the known effects to be guarded against’, where RP aimed to avoid the dangers of overexposure. In the 1950 Recommendations [11], superficial injuries, general effects on the body (particularly the blood and blood-forming organs), malignant tumors, other deleterious effects (e.g. cataracts, obesity, impaired fertility, and reduction of lifespan) and genetic effects were listed as ‘the effects to be considered’. RP, as described in ICRP-1 [12], aimed to prevent or minimize the ‘somatic effects’ that occur in an exposed individual (e.g. cataracts, leukemia and other malignant disease, impaired fertility, and life shortening), and to prevent deterioration of ‘genetic effects’ that occur in the offspring of exposed individuals. RP, as described in ICRP-9 [13], aimed to prevent the ‘acute effects’ that occur within a few weeks after exposure, to limit the risks of ‘late effects’ with a latent period of decades to an acceptable level, and to limit the ‘somatic effects’ in an exposed individual (e.g. cataracts, leukemia and other malignant disease, skin damage, impaired fertility and non-specific aging) and ‘hereditary effects’ (displacing ‘genetic effects’ used since ICRP-1) in the descendants of the exposed individual. ICRP-14 [14] defined three forms of the dose–response relationship: with no threshold, with a well-defined threshold, and with a quasi-threshold. ICRP-26 [15] was the first set of guidelines to classify radiation effects based on whether or not effects are stochastic, and this framework has heretofore been the cornerstone by which to consider dose limits for various radiation effects.

### Stochastic effects

ICRP-26 defined ‘stochastic effects’, where ‘stochastic’ was used to mean ‘of a random or statistical nature’. For an effect to be called stochastic, the probability of it occurring, but not its severity, was regarded as a function of dose without threshold. It recommended that RP aim at limiting the probability of the stochastic effects to an acceptable level, and an LNT dose–response relationship was assumed. Since ICRP-26, there have been no changes to the name or definition of stochastic effects, and only ‘cancer' and ‘hereditary effects’ (replacing ‘heritable effects’ used from ICRP-9) have been assigned to this category. Since ICRP-41 [16], stochastic effects have been supposed to result from injury to a single cell or small number of cells. ICRP-103 stated that RP aims to reduce risks of stochastic effects to the extent reasonably achievable, and that stochastic effects are either cancer development in exposed individuals owing to mutation of somatic cells or heritable disease in their offspring owing to mutation of reproductive cells.

### Tissue reactions

ICRP-26 defined ‘non-stochastic effects’ as those in which the probability and severity of an effect varying with dose and threshold may occur. RP was recommended so as to prevent detrimental non-stochastic effects, such as cataracts, non-malignant skin damage and impaired fertility. ICRP-41 defined ‘non-stochastic effects’ as damage resulting from the collective injury of substantial numbers or proportions of cells in affected tissues. It was considered that if the severity depends on the number or proportion of damaged cells, thresholds depend on the sensitivity of methods for detecting the damage. It was also considered that the time at which an effect may be detected depends on the temporal course of the injury, which varies depending on the extent to which the underlying damage is either repaired or progresses with time after irradiation.

ICRP-60 [17] proposed that because radiation-induced cell killing is a stochastic process, the term ‘non-stochastic effects’ is unsuitable for injury resulting from the death of a large number of cells. It was further thought that although the initial cellular changes are random, the large number of cells involved in the initiation of a clinically observable non-stochastic effect gives the effect a deterministic character. Thus, ‘non-stochastic’ was replaced with ‘deterministic’, defined to mean ‘causally determined by preceding events’.

ICRP-103 used the terms ‘harmful tissue reactions’ together with ‘deterministic effects’, because these effects were thought to be modifiable by post-irradiation procedures. It was mentioned that these effects are largely due to the killing or malfunction of cells following high-dose irradiation. It was considered that early tissue reactions (days to weeks) in cases where the threshold dose has been exceeded may be of the inflammatory type resulting from the release of cellular factors, or may be reactions resulting from cell loss.

ICRP-118 [4] referred to deterministic effects as tissue reactions because it was recognized that these effects are not predetermined at the time of irradiation and can be altered by the use of various biological response modifiers. Tissue reactions were defined as injury in populations of cells characterized by a threshold dose and an increase in the severity of the reaction as the dose is increased further. Acute doses of up to ∼0.1 Gy were believed to produce no functional impairment of tissues. Supplementary Table 1 recapitulates further details of changes in radiation effect classification.

## CHANGES IN DOSE LIMITATION CONCEPT

Occupational dose limits and public ones were first recommended in the 1934 Recommendations [18] and the 1954 Recommendations [19], respectively. The following three dose limitation concepts were used.

(i) ‘Tolerance dose’ was put forth in the 1934 Recommendations. This concept implicitly assumed a safe threshold below which no untoward effects would occur, because the term was coined to describe the entirely safe dose that a worker could tolerate without ultimately suffering injury [20]. Tolerance dose was recommended as a daily or weekly limit for external photon exposure of workers, and was expressed in ‘r’ (see Supplementary Table 2 for changes in dose units). There is no simple conversion formula from ‘r’ to ‘rad’ or ‘Gy’, but for simplicity we regard herein 1 ‘r’ as corresponding to 1 ‘rad’ and 1 ‘cGy’.

(ii) ‘Maximum permissible dose’ (MPD) displaced the tolerance dose in the 1950 Recommendations. MPD assumed no threshold, emerging experimental evidence for non-threshold-type effects (e.g. genetic effects) being taken into account. MPD was recommended as a weekly, quarterly or yearly limit for external and internal exposure of workers and the public to various types of radiation in addition to photons, and was expressed in ‘rem’ or ‘r’. The ‘rem’ is a singly weighted tissue dose or dose equivalent, where dose in ‘rem’ is produced by multiplying dose in ‘rad’ by relative biological efficiency or effectiveness (RBE), quality factor (QF or Q), dose distribution factor, and/or other necessary modifying factors (see Supplementary Table 3 for changes in radiation weighting factors).

(iii) ‘Dose limit’ or ‘dose equivalent limit’ replaced MPD in ICRP-9 for the public and in ICRP-26 for workers. Whilst tolerance dose and MPD largely relied on the skin erythema dose, dose limits were determined according to detriment (harm to health) for stochastic effects and according to threshold dose for tissue reactions. Dose limits were recommended as annual limits for external and internal exposure of workers and the public to all types of radiation, and were expressed in ‘rem’ or ‘Sv’. ‘Equivalent dose’ (formerly termed ‘dose equivalent’) is the sum of dose in Gy in a tissue or organ multiplied by a radiation-weighting factor (*w*_R_, formerly termed QF or Q) for all types of radiation. ‘Effective dose’ (formerly termed ‘dose equivalent’ or ‘effective dose equivalent’) is the sum of equivalent dose multiplied by a tissue-weighting factor (*w*_T_) in all specified tissues and organs. Currently, effective dose limits for the whole body and equivalent dose limits for the lens, skin, hands and feet have been recommended, all of which are expressed in ‘mSv/year’. Concepts of maintaining exposure have also evolved (see Supplementary Table 4).

## CHANGES IN DEFINITION OF DETRIMENT AND THRESHOLD

### Detriment

The detriment can be defined as the total harm to health experienced by an exposed group and its descendants as a result of the group's exposure to a radiation source, and allows for the variations in radio-sensitivity of different organs and tissues to the induction of stochastic effects [3]. The *w*_T_ represents the relative contribution of a tissue or organ in question to the total detriment following whole-body exposure, and is calculated by rounding off the relative detriment [3].

In the 1950s–1970s, ‘critical’ organs or tissues were assigned for whole-body exposure (the blood-forming organs assigned in 1950–1977, gonads in 1954–1977, lens in 1954–1964, and skin in 1954–1958). This was because these organs were considered particularly vulnerable and thereby critical from the RP viewpoint, and because the extension of occupational exposure to various types of radiation besides photons necessitated consideration of the dose in each organ. However, ICRP-14 pointed out that the critical organ concept does not allow for summation of the total health risks according to the relative sensitivity of the irradiated tissues. ICRP-14 further suggested that the relative sensitivity of various tissues could be properly compared only if a common scale was adopted for effects as diverse as cataracts, impaired fertility, tumor induction and genetic effects. It was also mentioned that not merely because there seems to be a sufficiently high threshold for cataract induction, but also because impaired fertility seems to be much less important than genetic defects in the first generation, the dose limits for parts of the body of workers (expressed as multiples of the dose limit for uniform whole-body irradiation) depend on (i) the relative importance of genetic damage and tumor induction after uniform whole-body irradiation, and (ii) the relative sensitivity of the part of the body to tumor induction. Thus, ICRP-14 would be the founding basis for the concept of effective dose limit. ICRP-14 also tentatively classified the relative radio-sensitivity of tissues and organs to cancer induction in adult life, and provided the risk factors for the induction of leukemia, bone tumors and thyroid tumors in a certain population. ICRP-8 [21] had previously provided the risk factors for the induction of leukemia and fatal somatic cancers for both sexes and all ages as well as the induction of juvenile childhood thyroid cancer according to the data of atomic bomb (A-bomb) survivors in Hiroshima and Nagasaki. The cancer induction risk factors presented in ICRP-8 and -14 were expressed in terms of cases per million per rad (i.e. 10^−4^ cases/Gy).

ICRP-26 mentioned that the risk factors for different tissues are based upon the estimated likelihood of inducing fatal malignant disease, non-stochastic changes, or substantial genetic defects expressed in live-born descendants, and listed various ‘tissues at risk’. Of these, the mortality risk factors were assigned in terms of fatal cancer, and the genetic risk factor was assigned for gonads in terms of hereditary effects. Skin and the lens were listed as tissues at risk in terms of cosmetically unacceptable changes and cataracts, respectively, but their risk factors were not assigned. Non-lethal cancer was omitted, but the 1980 Brighton Statement [22] crudely assessed detriment attributable to non-lethal cancer as ∼10% of the fatality detriment. ICRP-60 stated that detriment must include estimates of fatal cancer and other deleterious effects, and that the main components of the detriment consist of the fatal cancer mortality risk in all relevant organs, expected life lost for fatal cancer in different organs allowing for latency, morbidity from non-fatal cancer, and serious hereditary effects. Non-fatal cancer was thus included, and the weighted coefficient for curable cancer was ∼20% of the fatality coefficient. ICRP-103 scored the reduction in QOL associated with living with a serious illness, and used the incidence risks in place of the mortality risks because the incidence data were thought to provide a more complete description of the cancer burden. The *w*_T_ applies to all individuals (i.e. workers and the public, including pregnant women, developing fetuses and children) for the assessment of effective dose. Supplementary Table 5 lists changes in risk coefficients, detriment, and tissue weighting factors.

As regards detriment and *w*_T_ for cancer effects, ICRP-26 assigned risk coefficients for six categories (bone marrow for leukemia, and bone, lung, thyroid, breast and remainder tissues for somatic cancer). The 1978 Stockholm Statement [23] subsequently added skin, and ICRP-60 added esophagus, stomach, colon, liver, ovary and bladder (a total of 13 categories: ICRP-103 did not change this).

An increased cancer death probability rate will not occur until after a minimum latent period after radiation exposure, and four different models were employed to describe the subsequent excess probability rate as a function of time. First, ICRP-26 used the simple, additive model (also termed the ‘absolute model’), where the excess probability rate is dependent on dose but independent of age. Second, ICRP-60 used the simple, multiplicative model (also termed the ‘relative model’), where the excess rate increases with age at the same rate as the background cancer rate. Last, ICRP-103 used both the excess relative risk (ERR) and excess absolute risk (EAR) models where ERR:EAR weights of 0:100% were assigned for breast and bone marrow, 100:0% for thyroid and skin, 30:70% for lung, and 50:50% for all others. ICRP-60 and -103 applied a DDREF of 2, except for leukemia where the linear–quadratic model for risk already accounted for the DDREF.

ICRP-26 simply calculated the *w*_T_ as the nominal mortality risk coefficients in each tissue relative to the total risk coefficients for stochastic effects (fatal cancer and severe hereditary effects), for which non-stochastic effects and curable non-fatal cancer were not counted. Risk coefficients were determined based on observations in A-bomb survivors, radiotherapy patients and miners, or by comparison with leukemia risks. The same values were given for the whole population and for workers. ICRP-60 used the nominal lifetime cancer mortality risk coefficients adjusted for lethality fraction and years of life lost (YLL) to determine detriment, and provided separate detriments for whole population (0–90 years at the time of exposure: ATB) and for workers (18–64 years ATB). The relative risk coefficients were determined for China, Japan, Puerto Rico, the UK and the USA, and the lethality fraction was based on the US data. ICRP-103 used nominal lifetime cancer incidence risk coefficients adjusted for the lethality fraction, QOL and YLL to determine detriment, and provided separate detriments for whole population (0–85 years ATB) and for workers (18–64 years ATB). For nominal lifetime cancer-incidence risk coefficients and the lethality fraction, the same US data were used as in ICRP-60. ICRP-26, -60 and -103 assigned the *w*_T_ for 6, 12 and 14 categories, respectively, in addition to the ‘remainder’ tissues. ICRP-60 and -103 determined the *w*_T_ based on detriment for whole population as well as for workers.

The remainder tissues were considered, for which tumorigenic effects of radiation were not explicitly evaluated as individual cancer sites and thus detailed radiation risk calculations were uninformative. ICRP-26 assigned the *w*_T_ of 0.06 for each of five highly irradiated but unspecified tissues (i.e. 0.30 in total). ICRP-60 listed adrenals, brain, upper large intestine (c.f. ‘lower large intestine’ used to mean colon), small intestine, kidney, muscle, pancreas, spleen, thymus and uterus as remainder tissues. ICRP-103 excluded brain and upper large intestine, and listed 14 tissues (adrenals, extrathoracic tissue, gallbladder, heart, kidneys, lymphatic nodes, muscle, oral mucosa, pancreas, prostate, small intestine, spleen, thymus and uterus/cervix). ICRP-103 also newly assigned the *w*_T_ of 0.01 for each of brain and salivary glands (by judging their slightly higher cancer risk than ‘remainder tissues’, albeit not specifically quantifiable) (Supplementary Table 5).

Regarding detriment and *w*_T_ for gonads (i.e. male testes and female ovaries), ICRP-26 considered hereditary effects in the first two generations only, with the harm after the first two generations being considered less relevant [22]. The 1980 Brighton Statement raised concerns about the total genetic harm, and ICRP-60 considered hereditary effects in all future generations, assuming that estimates used in the risk equation will remain valid for tens or hundreds of human generations, and that the population structure, demography and health care facilities will remain constant over many centuries. ICRP-103 subsequently regarded those assumptions as very unrealistic and untestable, and hence considered heritable effects only up to the second generation. The *w*_T_ for gonads included detriment for ovaries in terms of cancer effects in ICRP-60 and -103, but not in ICRP-26.

### Threshold

ICRP-14 first described the threshold-type dose response, and ICRP-26 described the non-stochastic effects as having a threshold; however, neither of these documents properly defined the term ‘threshold’ itself. ICRP-41 defined a ‘practical’ threshold for RP purposes as the amount of radiation required to cause a particular effect in ‘at least 1–5%’ of exposed individuals. The number ‘1–5%’ appears to originate from the proposal by Rubin and Casarett [24], who defined a clinically acceptable minimum injurious tolerance dose, as a dose that causes severe complications in ‘1–5%’ of patients within 5 years post-radiotherapy. ICRP-103 changed the definition of threshold to become the amount of radiation required to cause a particular effect in ‘only 1%’ of exposed individuals. The level of 1% was considered optimal, not only because the use of a smaller level would entail a greater extrapolation to lower doses with concomitant greater uncertainties, but also because the use of a higher level would have less uncertainties but may not be acceptable for some endpoints. Note that the longer the follow-up period, the lower the threshold dose.

## CURRENT SITUATION

For nearly four decades, radiation effects have been classified depending on whether effects are stochastic. Dose limits have been recommended with this framework, in which effective dose limits aim to keep stochastic effects below unacceptable levels and equivalent dose limits aim to avoid tissue reactions. Supplementary Tables 6–8 summarize the changes in dose limits for planned exposure, and Supplementary Figure 1 plots dose limits for the whole body and lens as a function of Anno Domini time. Supplementary Table 9 summarizes the key papers that affected the decision of ICRP on the dose limit for lenses, and Supplementary Table 10 summarizes the key descriptions regarding the lens in ICRP publications.

**Fig 1.**
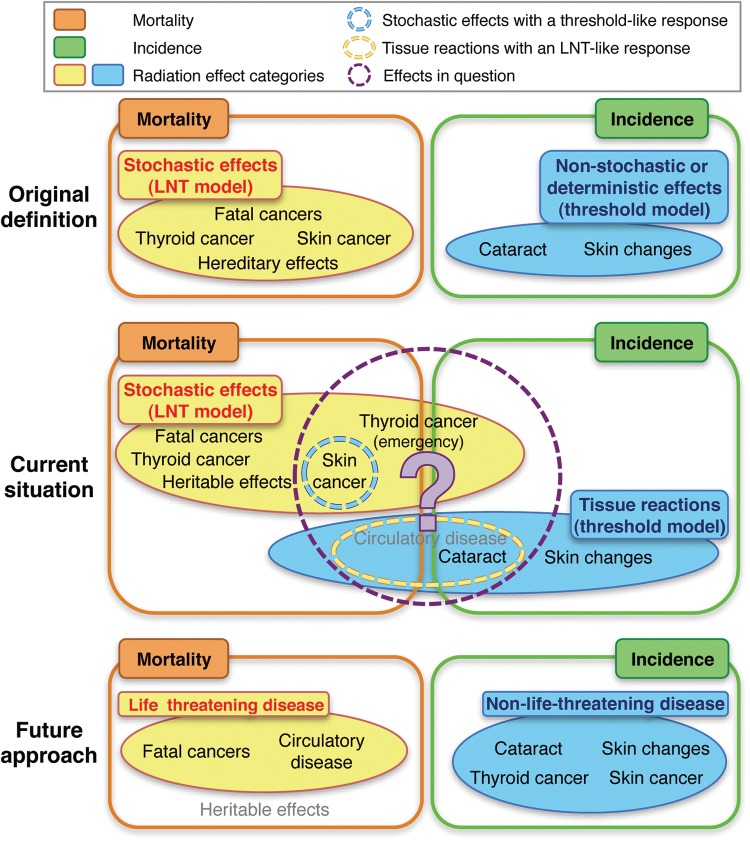
A diagram schematizing the original definition, current situation and our proposal for radiation effect classification and associated radiation protection (RP) endpoints. Orange- and green-colored solid lines indicate radiation effects of which risk is managed in terms of mortality and incidence, respectively. Yellow- and blue-colored solid areas represent radiation effect categories. The yellow-colored dotted line shows radiation effects categorized in tissue reactions but with a linear-non-threshold (LNT)-like dose response, whereas the blue-colored dotted line shows radiation effects categorized in stochastic effects but with a threshold-like dose response. A purple-colored dotted line further emphasizes radiation effects that obscure the boundary between stochastic effects and tissue reactions. Radiation effects were originally classified into stochastic effects and non-stochastic or deterministic effects (presently called tissue reactions). Stochastic effects comprise fatal cancer, non-fatal cancer (e.g. thyroid cancer and non-melanoma skin cancer) and fatal hereditary effects with an LNT dose–response model, whilst a threshold-like dose–response relationship has been known for several cancers such as skin cancer and bone cancer. Non-stochastic or deterministic effects include radiation effects with a threshold-type dose–response model (e.g. vision-impairing cataracts and cosmetically unacceptable non-cancer skin changes). For RP purposes, mortality and incidence were endpoints employed to manage the risk of stochastic effects and non-stochastic or deterministic effects, respectively, whereas an ‘emergency dose level’ for the thyroid was assigned based on its cancer incidence. At present, these have not been changed basically. However, a tissue reaction category now includes life-threatening circulatory disease, though its dose limit has not been set. Moreover, for cataracts and circulatory disease, epidemiological evidence now suggests an LNT dose response, and the RP system assumes no dose-rate effect, making the boundary between stochastic effects and tissue reactions fuzzy. Such a situation may be improved if radiation effects are classified into life-threatening disease and non-life-threatening disease, for which the risk management endpoints are mortality and incidence, respectively. Taken together, the necessity to limit heritable effects should also be reconsidered because human evidence has thus far shown no detectable transgenerational effects. This approach may be useful for future discussion.

In contrast to this framework, we are currently facing a situation that makes the boundary between stochastic effects and tissue reactions obscure (Figure 1), as discussed below.

### Stochastic effects with threshold?

A non-linear function of dose has been reported for several types of human cancer. For bone sarcoma in A-bomb survivors, a threshold of 0.85 Gy [95% confidence interval (CI): 0.12, 1.85 Gy] existed, above which there was a linear dose–response relationship [9]. For non-melanoma skin cancer in A-bomb survivors, the ‘spline’ model with a change in slope at 1 Gy fit the data better than a pure linear model [10]. *In vivo* experimental evidence that supports the existence of a threshold for skin cancer has long been discussed [25]. If a certain effect is classified as a stochastic effect but shows a threshold-type dose response, LNT-based dose limitation would overestimate the risk at a dose below the threshold.

### Tissue reactions with no threshold?

An LNT function of dose has been reported for several types of tissue reactions in humans. If a certain effect is classified as a tissue reaction but exhibits an LNT dose response, a threshold-based dose limitation would eventuate in risk underestimation at a dose below the threshold.

Ever since ICRP-14 defined a threshold-type dose–response relationship for detectable opacities based on three papers (Supplementary Table 9), generations of students have been taught that cataract is a typical tissue reaction with an apparent threshold. Nevertheless, mounting epidemiological evidence has challenged this long-standing tenet. A-bomb papers published in 1982 and 1990 [26, 27] first suggested an LNT dose response without threshold. ICRP-41 and -60 used these two papers as supporting references for an acute threshold for detectable opacities of 0.5–2 Gy, but ICRP-92 [28] questioned the existence of a threshold. Then, A-bomb papers published in 2006 and 2007 [5, 6] suggested a zero threshold, and ICRP-118 determined an acute threshold for VIC of 0.5 Gy using these two papers as supporting references. ICRP-118 concluded that cataract is a tissue reaction with a threshold albeit small because there was no direct evidence that a single damaged progenitor lens epithelial cell could produce a cataract, but it was the first ICRP document to discuss the possibility that cataract is a stochastic effect.

ICRP-118 included circulatory disease as a tissue reaction, the risk of which has not been included in the computation of the current dose limits. So, how to management of fatal circulatory disease risk would be a subject for discussion. One A-bomb paper published in 2010 [7] concluded the lack of threshold for CVD and CeVD, and was most influential in ICRP-118 recommending a threshold of 0.5 Gy for such circulatory disease. Likewise, two Mayak papers are also in support of the LNT dose response for CVD and CeVD [29, 30].

### What are the ‘target’ organs or tissues for circulatory disease?

Current equivalent dose limits have each been assigned to a particular organ or tissue to avoid a specific tissue reaction, i.e., in skin, hands and feet (with respect to cosmetically unacceptable skin non-cancer changes), and the lens (with respect to VIC). In this regard, which organs or tissues should be considered for RP to prevent circulatory disease? For instance, both A-bomb data [31] and *in vivo* experimental data [32] have suggested that radiogenic renal dysfunction contributes to the acceleration of CVD, raising a question as to whether adequate protection of the heart and brain are enough to prevent radiation-induced CVD and CeVD, or whether the kidneys and even the whole vasculature network should also be included. ICRP-103 assigned the *w*_T_ of 0.12 for 14 remainder tissues including the heart and kidney, so the current computation considers certain stochastic effects on these tissues but not circulatory disease: the same holds true for brain (its *w*_T_ of 0.01 considers only brain tumor) [3].

### Cancer vs non-life-threatening tissue reactions vs life-threatening tissue reactions

Current equivalent dose limits have each been assigned to avoid a non-life-threatening tissue reaction based on the threshold for its 1% incidence (i.e. 20 Gy for cosmetically unacceptable skin changes, and 0.5 Gy for VIC). For example, most cataracts are curable through ambulatory surgery; notwithstanding, cataracts should be prevented because they limit occupational performance and interfere with daily life activities. Conversely, the threshold of 0.5 Gy for the heart and brain is predicated on the 1% mortality with >10 years follow-up to prevent life-threatening circulatory disease. A question arises as to whether the 1% mortality dose for life-threatening circulatory disease is suitable to consider the dose limit, similarly to the 1% incidence dose for non-life-threatening cataracts and skin changes, although all of these are classified as tissue reactions.

By definition, the new occupational equivalent dose limit for a lens of 20 mSv/year aims to prevent VIC at the incidence of 1%/0.5 Sv (with a mortality of 0%/0.5 Sv) with >20 years follow-up. For comparison, the occupational effective dose limit of 20 mSv/year aims to reduce lifetime cancer risks to a mortality of 2.06%/0.5 Sv (and an incidence of 5.84%/0.5 Sv) (n.b. excluding the contributions of heritable effects) [3, 33]. For instance, exposure of the Japanese population to 0.5 Sv is estimated to increase VIC incidence from 75 to 76%, cancer incidence from 48 to 54%, and cancer mortality from 20 to 22% [33]. Thus, dose limits deal with cataract incidence (and, potentially, circulatory disease mortality) more strictly than cancer mortality, necessitating the development of a common scale with which to compare diverse effects, regardless of their life-threatening or stochastic nature.

### Mortality or incidence of thyroid cancer?

ICRP-14 pointed out that the reason for different radio-sensitivities to thyroid cancer induction in different human populations is not established, and that any firm conclusion about changes in sensitivity with age depends on excluding one or other of the available pieces of evidence for sensitivity in childhood. Despite four-plus decades since then, the thyroid cancer risk following adult exposure remains a subject of discussion [34]. Inclusion of the thyroid dose in the computation of occupational effective dose limit would be unnecessary if there waere no thyroid cancer risk for adults.

The thyroid is the exceptional tissue with the intervention level of averted or avertable equivalent dose for emergency exposure, considering its cancer incidence posed by radioiodine intake (the levels of dose for abnormal or emergency exposures are listed in Supplementary Table 11). A primary public health concern following the Fukushima nuclear accident [35–37] has been the thyroid cancer risk [38], because its incidence has attracted more attention than its mortality.

### Detriment for tissue reactions?

By current definition, detriment assumes an LNT dose response, and is only applicable to stochastic effects [3]. Notwithstanding, it would be crucial to recall discussions made decades ago on detriment from tissue reactions. ICRP-14 first proposed the necessity of a common scale for ‘all’ radiation effects, and this was repeated in ICRP-26. Nonetheless, the first detriment recommended in ICRP-26 did not include tissue reactions in the computation of *w*_T_ and effective dose, because of the sufficiently high threshold for cataract and the much lower importance given to impaired fertility than to genetic defects: in actuality, the thresholds for cataracts of 15 Sv recommended in 1977–1984, and >8 Gy in 1984–2011 were definitely high. Taken together, discussions were made during the preparation of ICRP-41, leading to the proposal that if the severity of non-stochastic effects can be scored (e.g. 0.05 for visual loss due to cataracts vs 1 for cancer deaths) in relation to incidence, then the RP policy may be shifted from the prevention of non-stochastic effects to their limitation [39]. However, ICRP-41 concluded that quantification of detriment from non-stochastic effects is not feasible: this was because the relation between severity and dose cannot be predicted precisely when the dose is accumulated at a low rate, and because effects such as cataract or ulcerative dermatitis cannot be equated with a disabling genetic disorder or fatal disease such as cancer. Then, ICRP-60 mentioned that it is implicit in the concept of detriment that the relevant doses are small, well below the thresholds for deterministic effects. Conversely, the 2011 Seoul Statement now recommended thresholds of 0.5 Gy for non-fatal cataracts and fatal circulatory disease, stimulating a resurgence of discussions on the computation of detriment from tissue reactions.

Using the aggregate ERR/Sv for circulatory disease risk, Little *et al*. [40] estimated the EAR/Sv (n.b. EAR corresponds to nominal risk) for all circulatory disease in nine countries, which ranged from 2.50%/Sv (95% CI: 0.77, 4.22 Sv) for France to 8.51%/Sv (95% CI: 4.00, 13.02 Sv) for Russia. The EAR/Sv for Japan was 4.01%/Sv (95% CI: 1.13, 6.89 Sv). In 2012, the Committee on Radiation Protection and Public Health of the Nuclear Energy Agency of the Organization for Economic Co-operation and Development (OECD/NEA/CRPPH) held the Third Workshop on Science and Values in Radiological Protection Decision Making (S&V workshop), where Schneider [41] presented his calculation of detriment for circulatory disease in the UK and French populations using risk coefficients from the A-bomb data [7] and the Mayak data (heart disease only). His estimated detriment was 0.6–2.4%/Sv when based on fatal effects and 0.8–3.1%/Sv when based on incidence [41]. Our calculation, using the risk coefficient from the A-bomb data [7] and the Japanese baseline mortality data, yielded ∼2.5%/Sv, which is of the same order as the numerical values calculated by Little [40] and Schneider [41]. The addition of such circulatory disease risk may not change the order of the risk of stochastic effects, given the notable uncertainties attached to applying a nominal value to a population, as computed by averaging over age groups and both sexes.

Regarding cataracts, Thorne [42] suggested the assignment of a *w*_T_ for the lens and inclusion of lens dose into the computation of effective dose, but did not make its calculation. We have here attempted to make the following calculation of detriment for cataracts by using the same approach as for cancers in ICRP-103. First, nominal risk coefficients for cancer were replaced with the incidence risk of lens opacification in the Japanese population [5]. Second, the lethality fraction (death equals one) for cancer was replaced with the fraction of an opacity progressing to a cataract with a certain degree of visual impairment (i.e. 1 when all opacities progress to visual loss due to cataract), and this fraction was conservatively estimated using the background incidence risk of opacities [43] and the rate of possessing a disability certificate [44] in the Japanese population. Third, the QOL factor for cancer that is a function of the lethality fraction was replaced with the time tradeoff utility values [45], where its value for visual loss was normalized to one. From these approaches, visual loss-adjusted nominal risk of cataract was calculated where visual loss was an endpoint, instead of lethality-adjusted nominal risk of cancer where death was an endpoint. Then, when the relative YLL for cancer was replaced with the relative years from manifestations of opacity to surgical intervention, detriment for cataract was estimated to be ∼1 × 10^−3^%/Sv. Detriment was calculated as ∼1%/Sv, given that all minor opacities progress to visual loss due to cataract, and such increases in detriment simply come from the increased fraction of opacity progressing to VIC from the order of 10^−3^ to 1. For comparison with detriment for cancer, visual loss was converted to the lethality fraction (given accidental death due to visual loss), and detriment of the order of as low as 10^−7^–10^−6^%/Sv was yielded. Thus, detriment for cataract can scarcely change total detriment. Incidentally, in our aforementioned calculation of detriment, the DDREF was set as 1, because new thresholds recommended for circulatory disease and cataract are 0.5 Gy, regardless of the rate of dose delivery [4].

## FUTURE PROSPECTS

### To be life threatening or not to be

The current situation discussed above somewhat raises the necessity of reconsidering the framework for radiation effect classification. For future discussion, we here propose one approach that divides radiation effects into life-threatening disease and non-life-threatening disease, for which mortality and incidence may be used as the RP endpoints, respectively (Figure 1). The current RP system employs effective dose that deals with the mortality risk for all cancers (i.e. fatal and non-fatal ones) and severe heritable effects. The lethality fraction in ICRP-103 for non-fatal cancers (0.07 for thyroid cancer and 0.002 for skin cancer) was one or two orders smaller than that for fatal cancers and fatal heritable effects, which ranged from 0.29–0.95. Interestingly, an emergency intervention level for the thyroid has been assigned in equivalent dose according to cancer incidence, and an equivalent dose limit for the skin has been assigned based on the incidence of cosmetically unacceptable non-cancer changes. Thus, the lethality fraction of 0.1 estimated in ICRP-103 can be taken as the boundary between life-threatening disease and non-life-threatening disease. Within this framework, the currently fuzzy boundary between stochastic effects and tissue reactions may be improved.

A common scale for all radiation effects should help us to compare the various effects, reminiscent of the discussion in ICRP-14. Equivalent dose limits for tissue reactions are based on the threshold. However, the acceptable level of each detriment and also that of the total detriment need to be considered, and the new concept of detriment needs to be elaborated if the dose limit for non-life-threatening disease is to be set based on detriment (this word does not necessarily mean detriment as currently defined). In contrast, it may not be so difficult to assign detriment for life-threatening disease (e.g. circulatory disease) by using the current approach for stochastic effects as described above. However, such calculations should be accompanied by a solid mechanistic explanation supporting the dose response. For circulatory disease, further evaluation of the mechanisms should precede quantification of its detriment.

One of the main conclusions of the Third S&V workshop was that ICRP should have a task group (TG) for considering the detriment associated with tissue reactions [46], who should take time to carefully deliberate the new concept of detriment. In view of this, ICRP Committees 1 and 2 will prepare a discussion paper on detriment. Taken together, TG91 approved in 2013 will prepare a position paper that reevaluates the necessity of DDREF [47], so that the change is possible in nominal risk coefficients.

### Other non-cancer effects?

In addition to cataract and circulatory disease, evidence has now emerged for increased risks of other radiogenic non-cancer effects. Glaucoma is not life threatening but causes irreversible visual field loss, which, unlike cataract, is incurable. Normal-tension glaucoma (but not other types of glaucoma) was associated with radiation exposure with an odds ratio of 1.31 at 1 Gy (95% CI: 1.11, 1.53) [48]. Taken together, associations between radiation exposure and life-threatening respiratory disease were found for pneumonia/influenza, but these appeared partially attributable to incident cancer and/or CVD [49]. These non-cancer effects will make the boundary between stochastic effects and tissue reactions more vague.

### Detriment from heritable effects?

The necessity of detriment for heritable effects should be reevaluated, especially in view of the discrepancies between experimental animal data and human data, where no effects have been identified in the offspring of irradiated humans, in marked contrast to those of irradiated animals [50, 51].

### Reference levels for therapy?

Equivalent dose limits have been set separately from effective dose limits, considering heterogeneous exposure. Such heterogeneous occupational exposure to the skin and lens is most frequent in clinical settings, including veterinary medicine. The dose to patients during diagnosis or therapy is clearly higher than that to medical workers. ICRP-73 [52] proposed a ‘diagnostic reference level’, but not a ‘therapeutic reference level’. Considering that the threshold dose to the lens, heart and brain is now 0.5 Gy, it might be necessary to consider a therapeutic reference level.

### Radiobiological research needs

So far, detriment for cancer and threshold for tissue reactions have been deduced from the human data. However, as ICRP-60 mentioned that a certain effects cannot justifiably be classified as tissue reactions or stochastic effects without knowledge of the mechanisms leading to the observable defects, more biological studies are clearly necessary to better understand the shape of the dose–response curve, persistency, latency and risks of effects/disease, along with plausible underlying mechanistic explanations. Future radiobiological research needs have recently been discussed in terms of DDREF, individual sensitivity, hormesis, systems biology, and use of tissue archives [53]. Thus, our present discussion only focuses on the three topics as outlined below.

(i) There is growing epidemiological evidence documenting an LNT dose response for cataracts and circulatory disease [5–7]. It is natural that as the longer the follow-up period becomes longer, the threshold dose becomes lower, and statistically, detection of a significant threshold becomes more difficult. It is also possible that early- and late-arising diseases result from different mechanisms. For instance, the human cataract data suggest that at an early time-point after exposure, posterior subcapsular cataract (the typical radiation cataract) occurs with an apparent threshold, but that at a late time-point, a threshold for posterior subcapsular cataract becomes less apparent, and instead cortical cataract (typical senile cataract) begins to emerge [26, 27, 54, 55]. It would thus be interesting to biologically test if early- and late-occurring cataracts exhibit a threshold-type dose response and an LNT dose response, respectively, and if this is the case, cataract can be both a tissue reaction and a stochastic effect.

(ii) Non-targeted effects can be broadly defined as the effects caused by spatio-temporal signal propagation from irradiated cells to neighboring cells and progeny cells [56], for which ample phenomenological evidence has been obtained with *in vitro* and *in vivo* experimental systems [57]. Such experimental studies have suggested that the non-targeted effects may either increase or decrease the induction of cancer and non-cancer disease (e.g. cataracts) in non-irradiated cells/tissues/organs [58–60]. Nonetheless, their relevance to human health is still unclear [61] and needs to be studied further. Also, the non-targeted effect concept may have the potential to necessitate a shift from the currently employed individual tissue- or organ-based approach for detriment, *w*_T_ and threshold to an individual disease-based approach.

(iii) RBE differs with the linear energy transfer (LET) of radiation, where high-LET radiation is more effective than low-LET radiation [62, 63]. The *w*_R_ that relates to stochastic effects (chromosome aberrations) has been used, without justification, for tissue reactions (without justification) to calculate an equivalent dose to the lens and skin. Taking cataracts as an example, ICRP-118 reduced the threshold for low-LET radiation in Gy, but the 2011 Seoul Statement lowered an equivalent dose limit in Sv. It should be noted that the lens is historically the only tissue for which ICRP recommended a special *w*_R_ in 1964–1977 (Supplementary Table 3), and that ICRP-92 could not recommend the *w*_R_ or RBE values for cataract due to a lack of human data and concern as to how to apply the experimental data (e.g. a high-LET RBE of several hundred [64, 65]—much higher than the commonly used *w*_R_ and RBE of ≤20). Thus, more studies are necessary in order to identify the biological mechanism whereby high-LET RBE becomes very high for cataract (e.g. due to enhanced cell killing/inactivation, abnormal differentiation, and/or excessive proliferation of lens epithelial cells).

## CLOSING REMARKS

Here we have reviewed the changes in radiation effect classification, dose limitation concepts, and definition of detriment and threshold. For RP purposes, ICRP-14 [14] defined a threshold-type dose–response relationship, and ICRP-26 divided radiation effects into stochastic effects or tissue reactions. Since then, effective dose limits and equivalent dose limits have been recommended within this framework. However, mounting epidemiological evidence for stochastic effects with a threshold-type dose response and tissue reactions with an LNT dose response has obscured the boundary between these two categories. In summary, the 2011 Seoul Statement stimulated discussion on various issues, e.g. the identification of target organs/tissues for circulatory disease, the computation of detriment for tissue reactions, and dose levels for the limitation of cancer risks vs the prevention of non-life-threatening tissue reactions vs the prevention of life-threatening tissue reactions. For future discussion, we have here proposed one approach that divides radiation effects into life-threatening disease and non-life-threatening disease, for which mortality and incidence may be used as the RP endpoints, respectively. Finally, we discussed emerging evidence for non-cancer effects other than cataracts and circulatory disease, questioned detriment for heritable effects, proposed therapeutic reference levels, and raised several areas of need for radiobiological research. Further mechanistic studies should provide a better understanding of the shape of the dose–response curve, persistency, latency and risks of effects/disease to plausibly explain epidemiological findings.

## SUPPLEMENTARY DATA

Supplementary data is available at the *Journal of Radiation Research* online.

Supplementary Data

## References

[1] Grubbé EH (1949). X-ray Treatment: its Origin, Birth, and Early History.

[2] ICRP (1928). International recommendations for X-ray and radium protection. Br J Radiol.

[3] ICRP (2007). The 2007 Recommendations of the International Commission on Radiological Protection. ICRP Publication 103. Ann ICRP.

[4] ICRP (2012). ICRP Statement on tissue reactions and early and late effects of radiation in normal tissues and organs—threshold doses for tissue reactions in a radiation protection context. ICRP Publication 118. Ann ICRP.

[5] Nakashima E, Neriishi K, Minamoto A (2006). A reanalysis of atomic-bomb cataract data, 2000–2002: a threshold analysis. Health Phys.

[6] Neriishi K, Nakashima E, Minamoto A (2007). Postoperative cataract cases among atomic bomb survivors: radiation dose response and threshold. Radiat Res.

[7] Shimizu Y, Kodama K, Nishi N (2010). Radiation exposure and circulatory disease risk: Hiroshima and Nagasaki atomic bomb survivor data, 1950–2003. BMJ.

[8] Hamada N, Fujimichi Y, Iwasaki T (2014). Emerging issues in radiogenic cataracts and cardiovascular disease. J Radiat Res.

[9] Samartzis D, Nishi N, Hayashi M (2011). Exposure to ionizing radiation and development of bone sarcoma: new insights based on atomic-bomb survivors of Hiroshima and Nagasaki. J Bone Joint Surg Am.

[10] Preston DL, Ron E, Tokuoka S (2007). Solid cancer incidence in atomic bomb survivors: 1958–1998. Radiat Res.

[11] ICRP (1951). International recommendations on radiological protection. Br J Radiol.

[12] ICRP (1958). *Recommendations of the International Commission on Radiological Protection*. ICRP Publication 1.

[13] ICRP (1966). *Recommendations of the International Commission on Radiological Protection*. ICRP Publication 9.

[14] ICRP (1969). Radiosensitivity and spatial distribution of dose. ICRP Publication 14.

[15] ICRP (1977). Recommendations of the International Commission on Radiological Protection. Ann ICRP.

[16] ICRP (1984). Non-stochastic effects of ionizing radiation. ICRP Publication 41. Ann ICRP.

[17] ICRP (1991). 1990 recommendations of the International Commission on Radiological Protection. ICRP Publication 60. Ann ICRP.

[18] ICRP (1934). International recommendations for X-ray and radium protection. Br J Radiol.

[19] ICRP (1995). Recommendations of the International Commission on Radiological Protection. Br J Radiol.

[20] Mutscheller A (1925). Physical standards of protection against roentgen-ray dangers. Am J Roentgenol Radiat Ther.

[21] ICRP (1966). The evaluation of risks from radiation. ICRP Publication 8. Health Phys.

[22] ICRP (1980). Statement and recommendations of the International Commission on Radiological Protection from its 1980 meeting. Br J Radiol.

[23] ICRP (1978). Statement from the 1978 Stockholm meeting of the ICRP. ICRP Publication 28. Ann ICRP.

[24] Rubin P, Casarett G (1972). A direction for clinical radiation pathology. The tolerance dose. Front Radiat Ther Oncol.

[25] Fry RJM (1989). The question of thresholds for carcinogenesis. Cancer Invest.

[26] Otake M, Schull WJ (1982). The relationship of gamma and neutron radiation to posterior lenticular opacities among atomic bomb survivors in Hiroshima and Nagasaki. Radiat Res.

[27] Otake M, Schull WJ (1990). Radiation-related posterior lenticular opacities in Hiroshima and Nagasaki atomic bomb survivors based on the DS86 dosimetry system. Radiat Res.

[28] ICRP (2003). Relative biological effectiveness (RBE), quality factor (*Q*), and radiation weighting factor (*w*_R_). ICRP Publication 92. Ann ICRP.

[29] Azizova TV, Muirhead CR, Moseeva MB (2012). Ischemic heart disease in nuclear workers first employed at the Mayak PA in 1948–1972. Health Phys.

[30] Azizova TV, Muirhead CR, Moseeva MB (2011). Cerebrovascular diseases in nuclear workers first employed at the Mayak PA in 1948–1972. Radiat Environ Biophys.

[31] Adams MJ, Grant EJ, Kodama K (2012). Radiation dose associated with renal failure mortality: a potential pathway to partially explain increased cardiovascular disease mortality observed after whole-body irradiation. Radiat Res.

[32] Lenarczyk M, Lam V, Jensen E (2013). Cardiac injury after 10 Gy total body irradiation: indirect role of effects on abdominal organs. Radiat Res.

[33] Fujimichi Y, Kosako T, Yoshida K (2013). Issues behind radiation protection of the ocular lens based on new dose limit. Jpn J Health Phys.

[34] Mabuchi K, Hatch M, Little MP (2013). Risk of thyroid cancer after adult radiation exposure: time to re-assess?. Radiat Res.

[35] Hamada N, Ogino H (2012). Food safety regulations: what we learned from the Fukushima nuclear accident. J Environ Radioact.

[36] Hamada N, Ogino H, Fujimichi Y (2012). Safety regulations of food and water implemented in the first year following the Fukushima nuclear accident. J Radiat Res.

[37] Merz S, Steinhauser G, Hamada N (2013). Anthropogenic radionuclides in Japanese food: environmental and legal implications. Environ Sci Technol.

[38] Tokonami S, Hosoda M, Akiba S (2012). Thyroid doses for evacuees from the Fukushima nuclear accident. Sci Rep.

[39] Matsudaira H (1981). The 1980 ICRP Committee 1 Rome meeting: Meeting report. Radiol Sci.

[40] Little MP, Azizova TV, Bazyka D (2012). Systematic review and meta-analysis of circulatory disease from exposure to low-level ionizing radiation and estimates of potential population mortality risks. Environ Health Perspect.

[41] Schneider T Potential impacts of circulatory diseases on the radiation health detriment: a first appraisal. http://www.cepn.asso.fr/en/publications/communications/110.

[42] Thorne MC (2012). Regulating exposure of the lens of the eye to ionizing radiations. J Radiol Prot.

[43] Sasaki H (2001). Cataract epidemiological study in different races and climatic conditions. J Jap Soc Cat Res.

[44] MHLW The result of the fact-finding investigation of disabled children and disabled persons in the fiscal year 2006. http://www.mhlw.go.jp/toukei/saikin/hw/shintai/06/dl/01_0001.pdf.

[45] Brown MM, Brown GC, Sharma S (2003). Health care economic analyses and value-based medicine. Surv Ophthalmol.

[46] Hendry J Topic 3. – Non-cancer effects. Summary of discussions. http://www.oecd-nea.org/rp/workshops/tokyo2012/topic3_breakout_summary.pdf.

[47] ICRP Task Group 91. Radiation risk inference at low-dose and low-dose rate exposure for radiological protection purposes. http://www.icrp.org/icrp_group.asp?id=83.

[48] Kiuchi Y, Yokoyama T, Takamatsu M (2013). Glaucoma in atomic bomb survivors. Radiat Res.

[49] Pham TM, Sakata R, Grant EJ (2013). Radiation exposure and the risk of mortality from noncancer respiratory diseases in the life span study, 1950–2005. Radiat Res.

[50] Tatsukawa Y, Cologne JB, Hsu WL (2013). Radiation risk of individual multifactorial diseases in offspring of the atomic-bomb survivors: a clinical health study. J Radiol Prot.

[51] Little MP, Goodhead DT, Bridges BA (2013). Evidence relevant to untargeted and transgenerational effects in the offspring of irradiated parents. Mutat Res.

[52] ICRP (1996). Radiological protection and safety in medicine. ICRP Publication 73. Ann ICRP.

[53] Morgan WF, Bair WJ (2013). Issues in low dose radiation biology: the controversy continues. A perspective. Radiat Res.

[54] Minamoto A, Taniguchi H, Yoshitani N (2004). Cataract in atomic bomb survivors. Int J Radiat Biol.

[55] Worgul BV, Kundiyev YI, Sergiyenko NM (2007). Cataracts among Chernobyl clean-up workers: implications regarding permissible eye exposures. Radiat Res.

[56] Hamada N (2014). What are the intracellular targets and intratissue target cells for radiation effects?. Radiat Res.

[57] Hamada N, Matsumoto H, Hara T (2007). Intercellular and intracellular signaling pathways mediating ionizing radiation-induced bystander effects. J Radiat Res.

[58] Mancuso M, Pasquali E, Leonardi S (2008). Oncogenic bystander radiation effects in *Patched* heterozygous mouse cerebellum. Proc Natl Acad Sci U S A..

[59] Worgul BV, Kleiman NJ, David JD A positive and a negative bystander effect influences cataract outcome in the irradiated lens. http://abstracts.iovs.org/cgi/content/abstract/46/5/832.

[60] Portess DI, Bauer G, Hill MA (2007). Low-dose irradiation of nontransformed cells stimulates the selective removal of precancerous cells via intercellular induction of apoptosis. Cancer Res.

[61] Hamada N, Maeda M, Otsuka K (2011). Signaling pathways underpinning the manifestations of ionizing radiation-induced bystander effects. Curr Mol Pharmacol.

[62] Hamada N (2009). Recent insights into the biological action of heavy-ion radiation. J Radiat Res.

[63] Hamada N, Imaoka T, Masunaga S (2010). Recent advances in the biology of heavy-ion cancer therapy. J Radiat Res.

[64] Brenner DJ, Medvedovsky C, Huang Y (1993). Accelerated heavy particles and the lens. VIII. Comparisons between the effects of acute low doses of iron ions (190 keV/µm) and argon ions (88 keV/µm). Radiat Res.

[65] Worgul BV, Medvedovsky C, Huang Y (1996). Quantitative assessment of the cataractogenic potential of very low doses of neutrons. Radiat Res.

