# Supplementary material for: Classification of radiation effects for dose limitation purposes: history, current situation and future prospects
Source: J Radiat Res. 2014 May 3;55(4):629–40. doi: 10.1093/jrr/rru019 (PMC4100010; doi:10.1093/jrr/rru019)
Supplement: Supplementary Data [file supp_55_4_629__index.html]

Classification of radiation effects for dose limitation purposes: history, current situation and future prospects — Classification of radiation effects for dose limitation purposes: history, current situation and future prospects — Supplementary Data 

# Classification of radiation effects for dose limitation purposes: history, current situation and future prospects

## Supplementary Data

Supplementary Data

**Files in this Data Supplement:**

- Supplementary Data - Pdf file
