# Supplementary material for: Classification of radiation effects for dose limitation purposes: history, current situation and future prospects
Source: J Radiat Res. 2014 May 3;55(4):629–40. doi: 10.1093/jrr/rru019 (PMC4100010; doi:10.1093/jrr/rru019)
Supplement: Supplementary Data [file supp_rru019_rru019supp.pdf]

Supplementary Information to "Classification of Radiation Effects: History, Current Situation and Future Prospects" by Nobuyuki Hamada and Yuki Fujimichi.

Total page numbers: 18 (including 1 figure, 29 references and 11 tables)

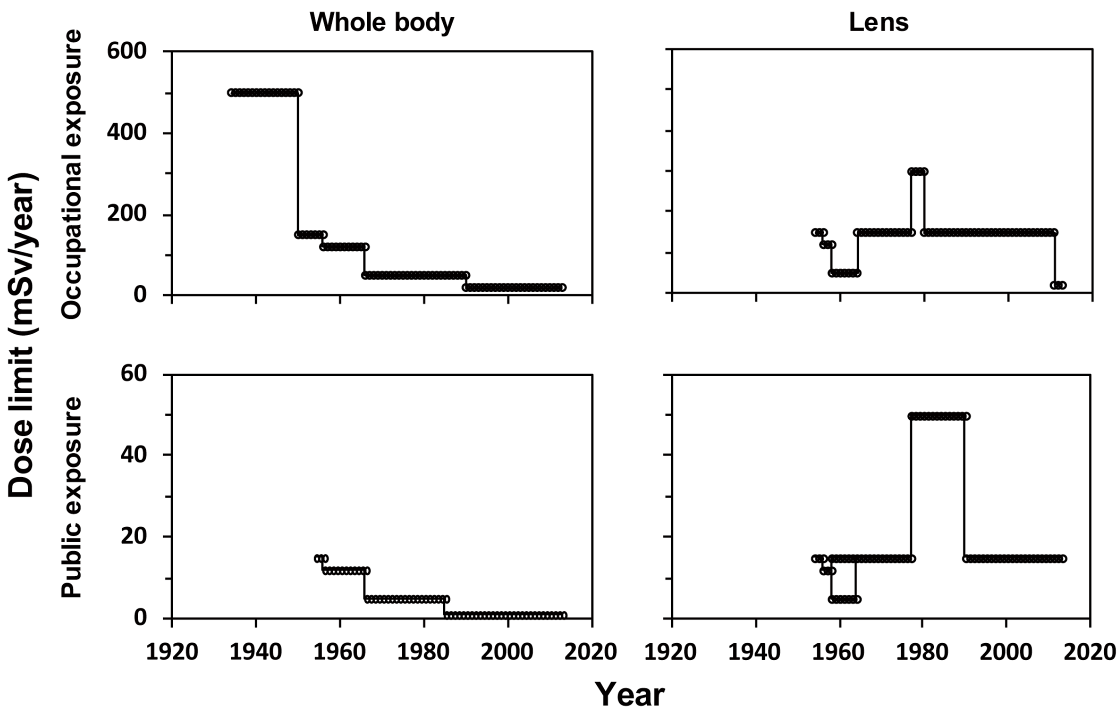

Supplementary Fig. 1. Alterations of dose limit as a function time. Upper panels, occupational limit. Lower panels, public limit. Left panels, whole body. Right panels, the lens. The data shown in Supplementary Tables 6-8 were plotted on a linear scale, where a daily, weekly or quarterly limit was converted into a yearly limit given a 5-day week, a 50-week year and a 4-quarter year.

SUPPLEMENTARY REFERENCES CITED IN SUPPLEMENTARY TABLES

S1. Preston DL, Ron E, Tokuoka S et al. Solid cancer incidence in atomic bomb survivors: 1958-1998. *Radiat Res* 2007;**168**:1-64.

S2. Cogan DG, Martin SF, Kimura SJ. Atom bomb cataracts. *Science* 1949;**110**:654-5.

S3. Abelson PH, Kruger PG. Cyclotron-induced radiation cataracts. *Science* 1949;**110**:655-7.

S4. Ham WT Jr. Radiation cataract. *Arch Ophthalmol* 1953;**50**:618-43.

S5. Cogan DG, Dreisler KK. Minimal amount of X-ray exposure causing lens opacities in the human eye. *Arch Ophthalmol* 1953;**50**:30-4.

S6. Britten MJA, Halnan KE, Meredith WJ. Radiation cataract – new evidence on radiation

dosage to the lens. *Br J Radiol* 1966;**39**:612-7.

S7. Merriam GR Jr, Focht EF. A clinical study of radiation cataracts and the relationship to dose. *Am J Roentgenol* 1957;**77**:759-85.

S8. Ham WT Jr. Fast neutron radiation hazards. In: Marion JB, Fowler JL (eds). Fast Neutron Physics. New York: Interscience Publishers, 1960, 841-927.

S9. Donaldson DD. Observations in Japan. In: Third conference on radiation cataracts: abstracts and proceedings. Washington, D.C.: National Research Council, National Academy of Sciences, 1952, 37-9.

S10. Cogan DG, Donaldson DD, Reese AB. Clinical and pathological characteristics of radiation cataract. *Arch Ophthalmol* 1952;**47**:55-70.

S11. Merriam GR Jr, Szechter A, Focht EF. The effects of ionizing radiation on the eye. *Front Radiat Ther Oncol* 1972;**6**:346-85.

S12. Morita K, Kawabe Y. Late effects on the eye of conformation radiotherapy for carcinoma of the paranasal sinuses and nasal cavity. *Radiology* 1979;**130**:227-32.

S13. Radiation effects in man: late or delayed effects. In: Langham WH (ed). Radiobiological factors in manned space flight. Washington, D.C.: Natl Acad Sci, Natl Res Council, 1967, 134-47.

S14. Otake M, Schull WJ. The relationship of gamma and neutron radiation to posterior lenticular opacities among atomic bomb survivors in Hiroshima and Nagasaki. *Radiat Res* 1982;**92**:574-95.

S15. Roth J, Brown N, Catterall M et al. Effects of fast neutrons on the eye. *Br J Ophthalmol* 1976;**60**:236-44.

S16. Otake M, Schull WJ. Radiation-related posterior lenticular opacities in Hiroshima and Nagasaki atomic bomb survivors based on the DS86 dosimetry system. *Radiat Res* 1990;**121**:3-13.

S17. NCRP. *Guidance on radiation received in space activities. NCRP Report No. 98*. New York: National Council on Radiation Protection and Measurements, 1989.

S18. Charles MW, Brown N. Dimensions of the human eye relevant to radiation protection. *Phys Med Biol* 1975;**20**:202-18.

S19. Shore RE, Worgul BV. Overview of the epidemiology of radiation cataracts. In: Junk AK,

Kundiev Y, Vitte P, Worgul BV (eds). Ocular radiation risk assessment in populations exposed to environmental radiation contamination. Dordrecht, The Netherlands: Kluwer Academic Publishers, 1999, 183-9.

S20. Albert RE, Omran AR, Brauer EW et al. Follow-up study of patients treated by X-ray epilation for *tinea capitis*. II. Results of clinical and laboratory examinations. *Arch Environ Health* 1968;**17**:919-34.

S21. Belkacémi Y, Ozsahin M, Pène F et al. Cataractogenesis after total body irradiation. *Int J Radiat Oncol Biol Phys* 1996;**35**:53-60.

S22. Nakashima E, Neriishi K, Minamoto A. A reanalysis of atomic-bomb cataract data, 2000–2002: a threshold analysis. *Health Phys* 2006;**90**:154-60.

S23. Neriishi K, Nakashima E, Minamoto A et al. Postoperative cataract cases among atomic bomb survivors: radiation dose response and threshold. *Radiat Res* 2007;**168**:404-8.

S24. Shore RE, Neriishi K, Nakashima E. Epidemiological studies of cataract risk at low to moderate radiation doses: (not) seeing is believing. *Radiat Res* 2010;**174**:889-94.

S25. Blakely EA, Kleiman NJ, Neriishi K et al. Radiation cataractogenesis: epidemiology and biology. *Radiat Res* 2010;**173**:709-17.

S26. Neriishi K, Nakashima E, Akahoshi M et al. Radiation dose and cataract surgery incidence in atomic bomb survivors, 1986-2005. *Radiology* 2012;**265**:167-74.

S27. Worgul BV, Kundiyev YI, Sergiyenko NM et al. Cataracts among Chernobyl clean-up workers: implications regarding permissible eye exposures. *Radiat Res* 2007;**167**:233-43.

S28. UNSCEAR. *Ionizing radiation: sources and biological effects. United Nations Scientific Committee on the Effects of Atomic Radiation 1982 report to the General Assembly, with annexes*. New York: United Nations, 1982.

S29. Minamoto A, Taniguchi H, Yoshitani N et al. Cataract in atomic bomb survivors. *Int J Radiat Biol* 2004:**80**:339-45.

Supplementary Table 1. Changes in radiation effect classification

ICRP recommended or reported in[a]	Classification
1928	The known effects to be guarded against: RP aims to avoid the dangers of overexposure<br>(1) injuries to the superficial tissues, (2) derangements of internal organs[b], and (3) changes in the blood
1950[c]	The effects to be considered<br>(1) superficial injuries, (2) general effects on the body, particularly the blood and blood-forming organs, e.g., production of anemia and leukemia, (3) the induction of malignant tumors, (4) other deleterious effects including cataract, obesity, impaired fertility, and reduction of life span, and (5) genetic effects
1958 (ICRP-1)	Somatic effects (occur in the exposed individual): RP aims to prevent or minimize these effects<br>e.g., cataract, leukemia and other malignant disease, impaired fertility, and shortening of life<br>Genetic effects (occur in the offspring of irradiated individuals): RP aims to prevent the deterioration of these effects
1966 (ICRP-9)[d]	Acute effects (occur within a few weeks after exposure): RP aims to prevent these effects<br>Late effects (with a latent period of tens of years): RP aims to limit the risks of these effects to an acceptable level<br>Somatic effects: RP aims to limit these effects in the exposed individual<br>e.g., cataract, leukemia and other malignant disease, skin damage, impaired fertility, and non-specific aging<br>Hereditary effects (affect the descendants of the exposed individual): RP aims to limit these effects in the whole population
1969 (ICRP-14)	Three forms of the dose-response relationship were defined<br>with no threshold (either linear or curvilinear), with a well-defined threshold, and with a quasi-threshold
1977 (ICRP-26)	Somatic effects (occur in the exposed individual) and hereditary effects (occur in the descendants of the exposed individual)<br>Stochastic effects (the probability of an effect occurring, and not its severity, is regarded as a function of dose without threshold): RP aims to limit the probability of stochastic effects to an acceptable level. Its limitation is achieved by keeping all justifiable exposures as low as reasonably achievable, economic and social factors being taken into account, subject always to the boundary condition that the appropriate dose equivalent limis shall not be exceeded. A linear-non-threshold dose-response relationship was assumed.<br>carcinogenesis (somatic effect), and hereditary effects<br>Nonstochastic somatic effects (the probalibity and severity of the effect vary with dose, and a threshold may occur): RP aims to prevent detrimental nonstochastic effects. Its prevention is achieved by setting dose equivalent limit at sufficiently low values.<br>e.g., cataract, non-malignant damage to the skin, and impaired fertility
1984 (ICRP-41)	Stochastic effects (may result from injury to a single cell or small number of cells)<br>Nonstochastic effects (damage resulting from the collective injury of substantial numbers or proportions of cells in affected tissues): if the severity depends on the number or proportion of damaged cells, the threshold dose will depend on the sensitivity of methods for detecting the damage. The time at which an effect may be detected will depend on the temporal course of the injury, and will vary depending on the extent to which the underlying damage is repaired or progresses with time after irradiation.
1990 (ICRP-58)	Stochastic effects (assumed to result from damage induced in single cells): severity is not dose-dependent, and the frequency increases with dose without a threshold.<br>Deterministic effects: detrimental effects that occur only after high doses (usually in excess of 1 Gy), and that are observed to be dose-dependent with respect to severity and frequency.
1990 (ICRP-60)	Stochastic effects: "stochastic" means "of a random or statistical nature". The clone of cells resulting from the reproduction of a modified but viable somatic cell may result, after the latency period, in the manifestation of a cancer. The probability of a cancer resulting from radiation usually increases with increments of dose, probably with no threshold, and in a way that is roughly proportional to dose, at least for doses well below the thresholds for deterministic effects. The severity of the cancer is not affected by the dose. If the damage occurs in a cell whose function is to transmit genetic information to later generations, any resulting effects, which may be of many different kinds and severity, are expressed in the progeny of the exposed person. This type of effect is called "hereditary".<br>Deterministic effects: "deterministic" means "causally determined by preceding events". If the number of cells lost is large enough, there will be observable harm reflecting a loss of tissue function. The probability of causing harm will be zero at small doses (some hundreds or thousands mSv depending on the tissue) and will increase steeply to unity above the threshold for clinical effect. The plot on linear axes of the probability of harm against dose is sigmoid. The severity of the harm increases with dose above clinical threshold. Although the initial cellular changes are random, the large number of cells involved in the initiation of a clinically observable, nonstochastic effect gives the effect a deterministic character.
2007 (ICRP-103)	Stochastic effects and cancer/heritable effects (RP aims to reduce risks to the extent reasonably achievable): either cancer development in exposed individuals owing to mutation of somatic cells or heritable disease in their offspring owing to mutation of reproductive (germ) cells. The probability of an effect occurring, but not its severity, is regarded as a function of dose without threshold.<br>Deterministic effects or harmful tissue reactions (RP aims to prevent these effects): injury in populations of cells, characterized by a threshold dose and an increase in the severity of the reaction as the dose is increased further. Modifiable by postirradiation procedures including biological response modifiers in some cases. Due in large part to the killing/malfunction of cells following high doses. Early tissue reactions (days to weeks) in cases where the threshold dose has been exceeded may be of the inflammatory type resulting from the release of cellular factors, or may be reactions resulting from cell loss. Late tissue reactions (months to years) are called “generic” and “consequential” if they arise as a result of “direct damage to the target tissue” and “early reactions”, respectively.<br>Noncancer diseases: somatic disease other than cancer (e.g., cardiovascular disease and cataracts).
2012 (ICRP-118)	Stochastic effects: malignant disease and heritable effects for which the probability of an effect occurring, but not its severity, is regarded as a function of dose without threshold.<br>Tissue reactions: injury in populations of cells, characterized by a threshold dose and an increase in the severity of the reaction as the dose is increased further. In some cases, these effects are modifiable by postirradiation procedures including biological response modifiers. Deterministic effects are preferably referred to as tissue reactions because it is increasingly recognized that these effects are not predetermined solely at the time of irradiation and can be altered after irradiation by the use of various biological response modifiers.

RP, radiation protection. [a]Reports are shown in italic fonts. [b]"particularly the generative organs" was added in the 1937 Recommendations. [c]Effects on the blood-forming organs were considered most dangerous to the wellbeing of the externally irradiated individual. [d]Carcinogenesis, degenerative effects such as cataract, developmental abnormalities in fetal tissue, and hereditary effects were listed as the most important effects, apart from the acute effects of large doses.

Supplementary Table 2. Changes in radiation dose units

**Category**	**SI derived unit**		**IXRPC or ICRP**	**IXUC or ICRU**	**Definition of unit dose and conversion into other units.**
	**Special name: symbol**	**Derived quantity (symbol)**	**started to use in**	**recommended in**	
Exposure dose	röntgen: r	N.A.	1934	1928 (Report 2)	1 esu in 1 cc of atmospheric air at 0°C and 76 cm of mercury pressure for X-rays
		N.A.	1937	1937 (Report 5)	1 esu in 1.293 mg of air for X- or γ-rays
		Exposure dose	1960 (ICRP-3)	1956 (Report 8)	*Ditto*
	röntgen: R	Exposure (*X*)	1970 (ICRP-15)	1962 (Report 10a)	1 R = 2.58 x 10^{-4} C/kg
Absorbed dose	erg/g	Energy absorbed	N.A.	1951 (Report 6)	Energy imparted to the material by ionizing particles at the place of interest.
	radiation absorbed dose: rad	Absorbed dose	1954	1953 (Report 7)	1 rad = 100 erg/g = 10 mGy = 0.01 J/kg
	gray: Gy	Absorbed dose (*D*)	1977 (ICRP-26)	1974	1 Gy = 1 J/kg = 100 rad
Singly weighted absorbed dose	gray-equivalent: Gy-Eq	RBE-weighted absorbed dose	2003 (ICRP-92)	N.A.	1 Gy-Eq = 1 Gy x RBE
	gray: Gy	RBE-weighted dose (RBE•*D*)	2007 (ICRP-103)	N.A.	1 Gy = 1 Gy x RBE
	gray-RBE: Gy(RBE)	RBE-weighted absorbed dose (D_{RBE})	N.A.	2007 (Report 78)	1 Gy(RBE) = 1 Gy x RBE
Singly weighted tissue dose	röntgen equivalent in man, mouse or mammal: rem	N.A.	1954	N.A.	1 rem = 1 rad x RBE = 10 mSv
		RBE dose	N.A.	1956 (Report 8)	*Ditto*
		Dose equivalent (*DE*)	1964 (ICRP-6)[a]	1962 (Report 10a)	1 rem = 1 rad x *QF* x *DF* x other necessary modifying factors
		Dose equivalent (*H*)	N.A.	1971 (Report 19)	*Ditto*
	sievert: Sv	Dose equivalent (*H*)	1977 (ICRP-26)[b]	1977 (Report 26)	1 Sv = 1 Gy x *Q* x *N* = 1 J/kg = 100 rem (*N* = 1)
		Equivalent dose	1990 (ICRP-60)[c]	1990	$H_T = \Sigma_R w_R D_{T,R}$
Doubly weighted dose	sievert: Sv	Dose equivalent	1977 (ICRP-26)[b]	1977 (Report 26)	$H = \Sigma_T w_T H_T$, $H_{wb,L}$, 1 Sv = 1 J/kg = 100 rem
		Effective dose equivalent	1978 (S.S.)	1993 (Report 51)	$H_E = \Sigma_T w_T H_T$
		Effective dose (*E*)	1990 (ICRP-60)[c]	1993 (Report 51)	*Ditto*

SI, Le Systèm International d'Unités. IXUC, International X-ray Unit Committee. ICRU, International Commission on Radiation Units and Measurements. N.A., not assigned. S.S., Stockholm Statement. RBE, relative biological effectiveness. *QF* and *Q*, quality factor. *DF*, dose distribution factor. *N*, other modifying factors (e.g., to take account of dose rate and fractionation). w_R, radiation weighting factor. $D_{T,R}$, mean absorbed dose from radiation R in a tissue or organ T. w_T, tissue weighting factor. $H_{wb,L,}$ dose equivalent for uniform irradiation of the whole body. esu, electrostatic unit. erg, ergon (1 erg = 10^{-7} J). [a]"other modifying factors" include a relative damage factor. For details, see ICRP-2 and -9. [b]Collective dose equivalent in a population (*S*), dose equivalent commitment (the infinite time integral of the *per caput* dose equivalent rate expressed as H_c) and committed dose equivalent (the dose equivalent accumulated over a working life of 50 years expressed as H_{50}) were also defined. *N* was actually set to 1 so that $H = DQN$ was changed to $H = DQ$ in ICRP-60 (but for operational quantities). [c]Committed dose (the equivalent dose and effective dose accumlated over 50 years for adults and up to age 70 for infants and children expressed as $H_T(\tau)$ and $E(\tau)$, respectively) and collective dose (effective dose *S* and equivalent dose S_T) were also defined.

Supplementary Table 3. Changes in radiation weighting factors

ICRP recommended in	Factors	Numerical values recommended						
		**1**	**1.7**	**2**	**5**	**8**	**10**	**20**
1950	Relative biological efficiency[a]	Radium γ-rays filtered by 0.5 mm Platinum, X-rays (0.1-3 MeV), β-rays	N.A.	N.A.	N.A.	N.A.	Protons and fast neutrons (<20 MeV)	α-rays
1954	RBE[b]	X-rays, electrons, positrons	N.A.	N.A.	N.A.	N.A.	Fast neutrons, protons (<10 MeV), naturally occurring α-particles	Heavy recoil nuclei
1964 (ICRP-6)	QF	X-rays, γ-rays, β^-, β^+	β^-, β^+, e^- (Emax ≤30 keV)	N.A.	N.A.	N.A.	Neutrons[c,d], α-particles	Heavy recoil nuclei, fission fragments, nuclei recoiling during α-emission
1966 (ICRP-9)	QF[e]	X-rays, γ-rays, β^-, β^+, conversion electrons	β^-, β^+, e^- (Emax ≤30 keV)	N.A.	N.A.	Neutrons from spontaneous fission	Neutrons, protons[c], α-particles	Heavy recoil nuclei, recoil fission fragments, accelerated heavy particles, fission fragments, nuclei recoiling during α-emission
1977 (ICRP-26)	$\bar{Q}$	X-rays, γ-rays, electrons	N.A.	N.A.	N.A.	N.A.	Neutrons, protons[f], single charged particles of unknown energy	α-particles, multiply charged particles of unknown charges/unknown energy
1985 (P.S.)	$\bar{Q}$	X-rays, γ-rays, electrons	N.A.	N.A.	N.A.	N.A.	Protons, single charged particles of unknown energy	Fast neutrons, α-particles, multiply charged particles of unknown charges/unknown energy
1990 (ICRP-60)	w_R[g]	Photons, electrons, muons (all energies)[h]	N.A.	N.A.	Neutrons (<0.01 MeV, >20 MeV), protons (>2 MeV)[i]	N.A.	Neutrons (0.01-0.1 MeV, 2-20 MeV)	Neutrons (0.1-2 MeV), α-particles, fission fragments, heavy nuclei
2007 (ICRP-103)	w_R[j]	Photons, electrons, muons	N.A.	Protons, charged pions	N.A.	N.A.	N.A.	α-particles, fission fragments, heavy ions

N.A., not accounted. RBE, relative biological effectiveness. QF or Q, quality factor. $\bar{Q}$, effective quality factor. Emax, maximum energy. P.S., Paris Statement. w_R, radiation weighting factor. [a]Defined as the inverse ratio of the dose of a given radiation to that of a reference radiation required to produce the same effect, assuming the same value for all effects of a given radiation except for gene mutations. [b]Defined as the appropriate value of the biological effectiveness of the radiation in question relative to that of a reference radiation, for the particular biological system and biological effect under consideration and for the condition under which the radiation is received. [c]QF values were given as a function of energy in terms of LET_∞ (the stopping power): QF = 1 for LET in water of ≤3.5 keV/μm, 1-2 for 3.5-7 keV/μm, 2-5 for 7-23 keV/μm, 5-10 for 23-53 keV/μm, and 10-20 for 53-175 keV/μm. [d]For the lens, a special QF of 30 shall be used in the case of high-LET particulate radiation, instead of the usual value of 10. [e]For the lens, an additional modifying factor of 1-3 was defined whose values change as a function of QF. For details, see the narrative for ICRP-9 in Supplementary Table 10. [f]Q values were given as a function of energy in terms of L_∞ (the collision stopping power): Q = 1 for LET in water of ≤3.5 keV/μm, 2 for 7 keV/μm, 5 for 23 keV/μm, 10 for 53 keV/μm, and 20 for ≤175 keV/μm. The effective quality factor was first defined in ICRP-15, and expressed as $\bar{Q}$ in ICRP-21. ICRP-21 listed $\bar{Q}$ values of 2-11 for neutrons (e.g., 2.3 for thermal neutrons) and 1.4-2.2 for protons. [g]All values relate to the radiation incident on the body or for internal sources, emitted from the source. For radiation types and energy that are not listed here, an approximation of w_R can be obtained by calculation of $\bar{Q}$ at a 10 mm depth in the ICRU sphere according to the equation presented in paragraph A14 of ICRP-60. [h]Excluding Auger electrons emitted from nuclei bound to DNA. [i]Protons other than recoil protons. [j]All values relate to the radiation incident on the body or for internal sources, emitted from the source. w_R for neutrons is given as a function of its energy (En). For En <1 MeV, $w_R = 2.5 + 18.2\exp\{-[\ln(En)]^2/6\}$. For 1 MeV≤ En ≤50 MeV, $w_R = 5.0 + 17.0\exp\{-[\ln(2En)]2/6\}$. For En >50 MeV, $w_R = 2.5 + 3.25\exp\{-[\ln(0.04En)]^2/6\}$.

Supplementary Table 4. Changes in concepts of maintaining exposure

ICRP recommended or reported in	Concepts of maintaining exposure
1928	The dangers of overexposure to X-rays and radium should be avoided
1950	Every effort should be made to reduce exposures to all types of ionizing radiations “to the lowest possible level”
1954	Exposure to radiation should be kept “at the lowest practicable level” in all cases (for workers)
1956	The accumlated dose should be kept “as low as practicable” (for workers especially up to age 30)
1958 (ICRP-1)	All doses should be kept “as low as practicable” and that any unnecessary exposure should be avoided (for workers and public)
1966 (ICRP-9)	All doses should be kept “as low as is readily achievable, economic and social considerations being taken into account”
1973 (ICRP-22)[a]	All doses should be kept “as low as is reasonably achievable, economic and social considerations being taken into account”
2007 (ICRP-103)	The likelihood of incurring exposure, the number of people exposed, and the magnitude of their individual doses should all be kept “as low as reasonably achievable, economic and societal factors being taken into account”

[a]Shown in italic fonts is a report.

Supplementary Table 5. Changes in risk coefficients, detriment, and tissue weighting factors

Effect Tissue	Whole population (0-90 years of age in ICRP-60, ages 0-85 years at the time of exposure in ICRP-103)														Working age population (18-64 years of age at the time of exposure)[a]				
	ICRP recommended in														ICRP recommended in				
	1977 (ICRP-26)				1990 (ICRP-60)					2007 (ICRP-103)					1977 (ICRP-26)	1990 (ICRP-60)		2007 (ICRP-103)	
	Fatal probability coefficient (10^{-4}/Sv)	Relative fatal probability coefficient	w_T	DDREF	Fatal probability coefficient (10^{-4}/Sv)	Detriment[b] (10^{-4}/Sv)	Relative detriment	w_T	DDREF	Fatal probability coefficient (10^{-4}/Sv)	Detriment[c] (10^{-4}/Sv)	Relative detriment	w_T	DDREF	Fatal probability coefficient (10^{-4}/Sv)	Fatal probability coefficient (10^{-4}/Sv)	Detriment[b] (10^{-4}/Sv)	Fatal probability coefficient (10^{-4}/Sv)	Detriment[c] (10^{-4}/Sv)
Cancer effect				N.A.					2										
Esophagus	N.A.	N.A.	N.A.		30	24.2	0.033	0.05		14	13.1	0.023	0.04	2	N.A.	24	19	15	14.2
Stomach	N.A.	N.A.	N.A.		110	100.0	0.138	0.12		66	67.7	0.118	0.12	2	N.A.	88	80	50	51.8
Colon	N.A.	N.A.	N.A.		85	102.7	0.142	0.12		31	47.9	0.083	0.12	2	N.A.	68	82	24	43.0
Liver	N.A.	N.A.	N.A.		15	15.8	0.022	0.05		29	26.6	0.046	0.04	2	N.A.	12	13	20	19.7
Lung	20	0.12	0.12		85	80.3	0.111	0.12		102	90.3	0.157	0.12	2	20	68	64	113	120.7
Bone	5	0.03	0.03		5	6.5	0.009	0.01		3	5.1	0.009	0.01	2	5	4	6	2	3.4
Skin	N.A.	N.A.	0.01[g]		2	4.0	0.006	0.01		2	4.0	0.007	0.01	2	N.A.	2	4	1	2.7
Breast	25	0.15	0.15		20	36.4	0.050	0.05		33	79.8	0.139	0.12	2	25	16	29	14	32.6
Ovary	N.A.	N.A.	N.A.		10	14.6	0.020	N.A.		6	9.9	0.017	N.A.	2	N.A.	8	12	4	6.6
Bladder	N.A.	N.A.	N.A.		30	29.4	0.041	0.05		13	16.7	0.029	0.04	2	N.A.	24	24	12	19.3
Thyroid	5	0.03	0.03		8	15.2	0.021	0.05		2	12.7	0.022	0.04	2	5	6	12	1	3.4
Bone marrow	20	0.12	0.12		50	104.0	0.143	0.12		28	61.5	0.107	0.12	N.A.[j]	20	40	83	15	23.9
Brain	N.A.	N.A.	N.A.		N.A.	N.A.	N.A.	N.A.		N.A.	N.A.	N.A.	0.01	2	N.A.	N.A.	N.A.	N.A.	N.A.
Salivary glands	N.A.	N.A.	N.A.		N.A.	N.A.	N.A.	N.A.		N.A.	N.A.	N.A.	0.01	2	N.A.	N.A.	N.A.	N.A.	N.A.
Other solid[d]	N.A.	N.A.	N.A.		N.A.	N.A.	N.A.	N.A.		71	113.5	0.198	N.A.	2	N.A.	N.A.	N.A.	43	65.4
Remainder tissues[e]	50	0.30	0.30		50	58.9	0.081	0.05		N.A.	N.A.	N.A.	0.12	2	50	40	47	N.A.	N.A.
Heritable effect[f]				N.A.					N.A.[i]					N.A.[i]					
Gonads	40	0.24	0.25		100	133.3	0.184	0.2[h]		16	25.4	0.044	0.08[h]		40	60	80	9.6	15.3
Total	165	1	1.01		600	725.3	1	1		414.6	574.2	1	1		165	460	555	324	422

For citation information, see the reference list in the supplementary information. N.A., not accounted. w_T, tissue weighting factor. DDREF, dose and dose-rate effectiveness factor. A-bomb, atomic bomb. [a]DDREF and w_T values are the same as those for the whole population. [b]The sex-averaged detriment for the whole population. The detriment of cancer was calculated as $\{F + k[(1 - k)F/k]\}l/15$, where F is the probability of fatal cancer, k is the lethality fraction, and $l/15$ is the relative length of life lost (the expected years of life lost for each fatal cancer divided by the average years of life lost for all cancers of 15 years). The detriment of severe genetic effect was calculated as [severe genetic effect (10^{-4}/Sv)]l/15. [c]The sex-averaged detriment for the whole population. The detriment of cancer was calculated as $\{Rk + R(1 - k)[(1 - q_{min})k + q_{min}]\}l$, where R is the nominal risk coefficient inclusive of the DDREF, k is the lethality fraction, l is the relative cancer-free life lost (n.b., l in ICRP-103 corresponds to $l/15$ in ICRP-60), and q_{min} is the minimum weight for nonfatal cancers (0 for the skin, 0.2 for the thyroid, and 0.1 for all other tissues or organs). [d]"Other solid" includes cancers of the small intestine, other parts of the digestive tract, the larynx, the thymus, the bone, connective tissues, genital organs (mela and female), the urothelial system (renal pelvis, ureter and urethra), the endocrine organs other than the thyroid, the oral cavity, the rectum, the gallbladder, the pancreas, the uterus, the prostate, the renal cell, the brain, and the central nervous system, nasal cavity tumors, melanomas, respiratory tract cancers, and male breast cancers according to the Life Span Study of the Japanese A-bomb survivors [S1] cited by ICRP-103, and contributed to w_T of the remainder tissues. [e]ICRP-26 and the 1978 Stockholm Statement did not concretely specify remainder tissues. In ICRP-26, w_T of 0.06 was assigned to each of the five most highly irradiated other organs and tissues. Remainder tissues listed in ICRP-60 were adrenals, brain, upper large intestine (c.f., "colon" was used to mean the lower large intestine), small intestine, kidney, muscle, pancreas, spleen, thymus and uterus. The remainder dose was defined in ICRP-60 by the mass-weighted average of the equivalent dose to those 10 remaider tissues or organs. In those exceptional cases in which a single one of the remainder tissues or organs receives an equivalent dose in excess of the highest dose in any of the twelve organs for which w_T is specified, w_T of 0.025 should be applied to that tissue or organ and w_T of 0.025 to the average dose in the rest of the remainder as defined above. In ICRP-103, brain and upper large intestine were excluded, and extrathoracic tissue, gallbladder, heart, lymphatic nodes, oral mucosa, and uterus/cervix were added. In ICRP-103, the equivalent dose to the 14 specified remainder tissues were added with no further mass weighting, meaning that w_T given to each remainder tissue is smaller than the least value assigned to any of the named tissues (i.e., 0.01). [f]ICRP-60 considered the heritable effects over all generations, whilst ICRP-26 and -103 considered these effects up to the first two generations. [g]The skin was added in the 1978 Stockholm Statement without indication of its corresponding mortality risk factor. [h]The w_T of gonads (heritable) includes detriment for ovaries, and is applied to the mean of the doses to testes and ovaries. [i]Unlike the case for cancer, the F and R of severe genetic effects were not derived from the A-bomb data, so that DDREF was not applied to heritable effects. [j]The linear-quadratic model for leukemia risk is inclusive of the DDREF.

Supplementary Table 6. Changes in dose limit for the lens

ICRP recommended in	Nomenclature	Scientific basis	Radiation workers		Members of the public	
			**Original values**	**Values in mSv/year**	**Original values**	**Values in mSv/year**
1954	Maximum permissible dose	Skin erythema dose	0.3 rem/week	150[j]	0.03 rem/week	15[j]
1956	Maximum permissible dose	Skin erythema dose	0.3 rem/week or 3 rem/13 weeks	120[k]	0.03 rem/week or 0.3 rem/13 weeks	12[k]
1958 (ICRP-1)	Maximum permissible dose	Skin erythema dose	*D* = 5(*N*-18)[a] or 3 rem/13 weeks (age ≥18)	50	1.5 rem/year[b]	15
			5 rem/year or 60 rem up to age 30 (age <18)		0.5 rem/year[c]	5
1964 (ICRP-6)	Maximum permissible dose	Skin erythema dose (ICRP-1)	4 rem/13 weeks or 15 rem/year	150	1.5 rem/year[b,d]	15
1966 (ICRP-9)	Maximum permissible dose	Skin erythema dose (ICRP-1)	15 rem/year	150		
	Dose limit	Skin erythema dose (ICRP-1)			1.5 rem/year	15
1977 (ICRP-26)	Dose equivalent limit[e]	Below a threshold dose[f]	300 mSv/year	300	50 mSv/year	50
1980 (B.S.)	Dose equivalent limit[e]	Skin erythema dose (ICRP-1)[g]	150 mSv/year	150	50 mSv/year	50
1990 (ICRP-60)	Equivalent dose limit	Below a threshold dose rate[h]	150 mSv/year	150	15 mSv/year	15
2007 (ICRP-103)	Equivalent dose limit	Below a threshold dose rate[h]	150 mSv/year	150	15 mSv/year	15
2011 (S.S.)	Equivalent dose limit	Preventive, implementable, optimized[i]	100 mSv/5 years, ≤50 mSv/year	20	15 mSv/year	15

B.S., Brighton Statement. S.S., Seoul Statement. [a]*D* and *N* are the accumulated tissue dose in rem and the age in years (*N* ≥18), respectively. [b]The value was assigned for individual adults who work in the vicinity of controlled areas or those who enter controlled areas occasionally. [c]The value was assigned for any individual of the public (i.e., including children and pregnant women) who resides in the neighborhood of controlled areas. [d]The value was assigned for any individual members of the population at large (including persons living in the neighborhood of controlled areas, and children). [e]The concept corresponds to the current definition of an equivalent dose limit. [f]Based on a threshold of >15 Sv (n.b., its supporting references were not provided). Occupational dose limit of 300 mSv/year was derived from 15 Sv (a dose that was judged as being below a threshold for the production of vision-impairing cataract by protracted exposure over the working lifetime of 50 years to low- or high-LET radiation) divided by 50 years. [g]The dose limit recommended in ICRP-9 was used, whereas Task Group suggested 100 mSv/year. [h]A threshold of >8 Sv reported in ICRP-41 was used. "8 Sv" was the number derived from 0.15 Sv/year x 50 years to explain the validity of 150 mSv/year, but not a threshold estimated to cause vision-impairing cataract in "at least 1-5%" or "1%" of exposed individuals. It can be considered that the dose limit of 150 mSv/year came from ICRP-1. [i]Based on a threshold for vision-imparing cataract of 0.5 Gy, an occupational limit of 20 mSv/year (but not 10 mSv/year) was recommended, along with a recommendation of optimization of protection for exposure of specific tissue. This was because a higher limit would not be protective given the substantially lower threshold, and because alignment with the effective dose limit (20 mSv/year) facilitates implementation. [j]A weekly value was converted into a yearly value given a 50-week year. [k]A yearly value was calculated by multiplying a quarterly value by 4.

Supplementary Table 7. Changes in occupational dose limits for the whole body, blood-forming organs, gonads, thyroid and bone

IXRPC or ICRP recommended in	Nomenclature (scientific basis)	Exposure types	Radiation workers: Whole body: Original values	Radiation workers: Whole body: Values in mSv/y	Blood-forming organs[a]. ED: 5 cm (critical organs: 1950-1977)	Gonads. ED: 7 cm (ovary) and ≤1 cm (testes) (critical organs: 1954-1977)	Thyroid	Bone
1934	Tolerance dose (SED)	External	0.2 r/d[b] (X-rays)	500[s]	N.A.	N.A.	N.A.	N.A.
1937	Tolerance dose (SED)	External	0.2 r/d[b] or 1 r/wk[b] (X- and γ-rays)	500[s]	N.A.	N.A.	N.A.	N.A.
1950	Maximum permissible dose (SED)	External	0.5 r/wk[b,d], 0.3 r/wk[c,d] or 1.5 r/wk[e]	150[t]	0.3 r/wk	N.A.	N.A.	N.A.
		Internal	0.3 r/wk		N.A.	N.A.	N.A.	N.A.
1954[f]	Maximum permissible dose (SED)	External	0.3 r/wk[c,g]	150[t]	0.3 rem/wk	0.3 rem/wk or 0.3 r/wk[g]	N.A.	N.A.
		Internal	0.3 rem/wk (critical organs)[k]		N.A.	N.A.	N.A.	0.06 rem/wk
1956[f]	Maximum permissible dose (SED)	External	0.3 r/wk or 3 r/13 wk	120[u]	0.3 rem/wk or 3 rem/13 wk	0.3 rem/wk or 3 rem/13 wk, 0.3 r/wk or 3 r/13 wk[g]	N.A.	N.A.
		Internal	0.1 rem/wk		N.A.	0.1 rem/wk	N.A.	N.A.
1958 (ICRP-1)[h]	Maximum permissible dose (SED)[i]	External/internal	N.A.		*D* = 5(*N*-18)[j] or 3 rem/13 wk (age ≥18)<br>5 rem/y or 60 rem up to age 30 (age <18)	*D* = 5(*N*-18)[j] or 3 rem/13 wk (age ≥18)<br>5 rem/y or 60 rem up to age 30 (age <18)	8 rem/13 wk<br>or 30 rem/y	N.A.
	Maximum permissible dose (SED)	Internal	3 rem/13 wk[l]	120[u]	N.A.	N.A.	N.A.	N.A.
1959[h]	Maximum permissible dose (SED)[i]	External/internal	N.A.		*D* = 5(*N*-18)[j] or 3 rem/13 wk (age ≥18)<br>5 rem/y or 60 rem up to age 30 (age <18)	*D* = 5(*N*-18)[j] or 3 rem/13 wk (age ≥18)<br>5 rem/y or 60 rem up to age 30 (age <18)	8 rem/13 wk<br>or 30 rem/y	N.A.
	Maximum permissible dose (SED)	Internal	3 rem/13 wk[l]	120[u]	N.A.	N.A.	N.A.	N.A.
1964 (ICRP-6)[h]	Maximum permissible dose (SED)[i]	External/internal	1.5(*N*-*A*) and 5(*N*-*B*)[m]		*D* = 5(*N*-18)[j] or 3 rem/13 wk (age ≥18)<br>5 rem/y or 60 rem up to age 30 (age <18)	*D* = 5(*N*-18)[j] or 3 rem/13 wk (age ≥18)<br>5 rem/y or 60 rem up to age 30 (age <18)	8 rem/13 wk<br>or 30 rem/y	8 rem/13 wk<br>or 30 rem/y
	Maximum permissible dose (SED)	Internal	3 rem/13 wk[l]	120[u]	N.A.	N.A.	N.A.	N.A.
1966 (ICRP-9)[h]	Maximum permissible dose (SED)[n]	External/internal	5 rem/y	50	5 rem/y[o]	5 rem/y[o]	30 rem/y	30 rem/y
1977 (ICRP-26)	Dose equivalent limit (detriment)[n,p]	External/internal	50 mSv/y[p]	50	500 mSv/y[q]	500 mSv/y[q]	500 mSv/y[q]	500 mSv/y[q]
1990 (ICRP-60)	Dose limit (detriment)	External/internal	20 mSv/y (100 mSv/5 y, ≤50 mSv/y)[r]	20	N.A.	N.A.	N.A.	N.A.
2007 (ICRP-103)	Dose limit (detriment)	External/internal	20 mSv/y (100 mSv/5 y, ≤50 mSv/y)[r]	20	N.A.	N.A.	N.A.	N.A.

N.A., not assigned. SED, skin erythema dose. ED, an assumed effective depth. d, day. wk, week. y, year. MPD, maximum permissible dose. [a]The term "blood-forming organs" was replaced with "red bone marrow" in ICRP-9. Then, "bone marrow" or "active bone marrow" have also been used along with "red bone marrow" since ICRP-28 and -30, respectively. [b]Surface dose (dose measured at the surface of the body). [c]Air dose (dose measured in free air). [d]Applicable to X- and γ-rays <3 MeV. [e]Applicable to high-energy β-rays, and energy equivalent to hard γ-rays. [f]The doses shown are additional to the natural background radiation. [g]Applicable to electromagnetic radiation <3 MeV. [h]The maximum permissible genetic dose of 5 rem/generation to the whole population (e.g., to exposure of radiation workers, special groups and population at large) was suggested. The term was changed to "genetic dose limit" in ICRP-9. [i]The doses shown are from all sources of internal and external radiation but exclude the dose from medical exposure and the natural background radiation. [j]*D* and *N* are the accumulated tissue dose in rem and the age in years (*N* ≥18), respectively. [k]The value was not given to specific organs, and the whole body and gastrointestinal tract were shown as example critical organs under some conditions. [l]Whole-body exposure from uptake of several radioisotopes. [m]This formula applies to the addition of doses from penetrating external exposure and from internal exposure in the case of long-lived bone-seeking radioisotopes. If the estimated dose body burden is >1/2 of MPD but <MPD, the whole body external exposure shall be limited to ≤1.5 rem/year. The total accumulated dose should not exceed 1.5(*N*-*A*) rem where *N* is the current age and *A* is the age when the body burden was first found to exceed 1/2 of MPD. If the body burden is found to have dropped to less than 1/2 of MPD at age *B*, the accumulated dose from external whole body exposure beyond age *B* should not exceed 5(*N*-*B*) rem. [n]The doses shown are from internal and external sources imposed by the occupation and exclude the dose from medical exposure, the natural background radition and other exposure received by the individual as a member of the public. [o]For flexibility, 2 rem/13 weeks and 5(*N*-18) were permitted for workers of age ≥18 where *N* is the age in years. Besides, 60 rem up to age 30 were permitted for workers of age <18. [p]A dose equivalent limit for the whole body (an effective dose limit in the modern term) was based on detriment, but not that for paricular organs. This was renamed "effective dose equivalent limit" by the 1978 Stockholm Statement. [q]A dose equivalent limit (an equivalent dose limit in the modern term) of 500 mSv/year was set for all organs other than the lens. [r]Effective dose limit. It is implicit that the dose constraint for optimization should not exceed 20 mSv/year. [s]A daily value was converted into a yearly value given a 5-day week and a 50-week year. For simplicity, 1 r was treated as 10 mSv. [t]A weekly value was converted into a yearly value given a 50-week year. For simplicity, 1 r was treated as 10 mSv. [u]A yearly value was calculated by multiplying a quarterly value by 4.

Supplementary Table 8. Changes in dose limits for occupational exposure (other than whole body, lens, blood-forming organs, gonads, thyroid and bone) and public exposure (other than the lens)

ICRP recommended in	Nomenclature (scientific basis)	Exposure types	Radiation workers						Members of the public
			Skin. ED: 7 mg/cm^2 (critical organs: 1954-1958)	Hands and forearms. ED: 7 mg/cm^2	Feet and ankles	Head and neck	Other organs[a]	Women of reproductive age or pregnant women	
1950	MPD (SED)	External	N.A.	1.5 r/wk[b]	N.A.	N.A.	N.A.	N.A.	N.A.
1954[c]	MPD (SED)	External	0.6 rem/wk (1.5 rem/wk[d])	1.5 rem/wk	1.5 rem/wk	1.5 rem/wk	BDD	N.A.	1/10 of the occupational limit
1956[c]	MPD (SED)	External	0.6 rem/wk or 6 rem/13 wk<br>(1.5 rem/wk or 15 rem/13 wk[d])	1.5 rem/wk or<br>15 rem/13 wk	1.5 rem/wk or<br>15 rem/13 wk	1.5 rem/wk or<br>15 rem/13 wk	N.A.	N.A.[e]	1/10 of the occupational limit
1958 (ICRP-1)	MPD (SED)[g]	External/internal	8 rem/13 wk or 30 rem/y	20 rem/13 wk<br>or 75 rem/y	20 rem/13 wk<br>or 75 rem/y	N.A.	4 rem/13 wk<br>or 15 rem/y	N.A.	1.5 rem/y for the blood-forming organs and gonads[h]<br>3 rem/y for the skin and thyroid[h]<br>0.5 rem/y for the blood-forming organs and gonads[i]
	MPD (SED)	Internal	N.A.	N.A.	N.A.	N.A.	N.A.	N.A.	1/10 of the occupational limit[k]<br>0.5 rem/y for the whole body[i]
1959	MPD (SED)[g]	External/internal	8 rem/13 wk or 30 rem/y	20 rem/13 wk<br>or 75 rem/y	20 rem/13 wk<br>or 75 rem/y	N.A.	4 rem/13 wk<br>or 15 rem/y	3 rem/13 wk[l]	1.5 rem/y for the blood-forming organs and gonads[h]<br>3 rem/y for the skin and thyroid[h]<br>0.5 rem/y for the blood-forming organs and gonads[i]
	MPD (SED)	Internal	N.A.	N.A.	N.A.	N.A.	N.A.	N.A.	1/10 of the occupational limit[k], 0.5 rem/y for the whole body[i]
1964 (ICRP-6)	MPD (SED)[g]	External/internal	8 rem/13 wk or 30 rem/y	20 rem/13 wk<br>or 75 rem/y	20 rem/13 wk<br>or 75 rem/y	4 rem/13 wk<br>or 15 rem/y	4 rem/13 wk<br>or 15 rem/y	1.3 rem/13 wk or 5 rem/y[m]<br>1 rem/pregnancy[n]	1.5 rem/y for the blood-forming organs and gonads[h]<br>0.5 rem/y for the blood-forming organs and gonads[o]<br>1/10 of the occupational limit for other organs[h,o]
	MPD (SED)	Internal	N.A.	N.A.	N.A.	N.A.	N.A.	N.A.	1/10 of the occupational limit[k], 0.5 rem/y for the whole body[i]
1966 (ICRP-9)	MPD (SED)[j]	External/internal	30 rem/y	75 rem/y	75 rem/y	N.A.	15 rem/y	1.3 rem/13 wk or 5 rem/y[m]<br>1 rem/pregnancy[n]	
	Dose limit (SED)	External/internal	N.A.	N.A.	N.A.	N.A.	N.A.	N.A.	1/10 of the occupational limit, 1.5 rem/y for the thyroid (age <16)
1977 (ICRP-26)	DEL (detriment)[j,p]	External/internal	500 mSv/y[q]	500 mSv/y[q]	500 mSv/y[q]	500 mSv/y[q]	500 mSv/y[q]	3/10 of the occupational<br>limit during pregnancy	5 mSv/y for the whole body[p]<br>50 mSv/y for the skin[f]
1985 (P.S.)	DEL (detriment)[j,p]	External/internal	500 mSv/y[q]	500 mSv/y[q]	500 mSv/y[q]	500 mSv/y[q]	500 mSv/y[q]	3/10 of the occupational<br>limit during pregnancy	1 mSv/y (70 mSv/70 years, ≤5 mSv/y) for the whole body[p]<br>50 mSv/y for the skin[f]
1990 (ICRP-60)	Dose limit (detriment)[p]	External/internal	500 mSv/y[r]	500 mSv/y[r]	500 mSv/y[r]	N.A.	N.A.	2 mSv/pregnancy	50 mSv/year for the skin[r], effective dose of 1 mSv/y (5 mSv/5 y)
		Internal	N.A.	N.A.	N.A.	N.A.	N.A.	About 1 mSv/pregnancy	
2007 (ICRP-103)	Dose limit (detriment)[p]	External/internal	500 mSv/y[r]	500 mSv/y[r]	500 mSv/y[r]	N.A.	N.A.	1 mSv to embryo/fetus	50 mSv/year for the skin[r], effective dose of 1 mSv/y (5 mSv/5 y)

N.A., not assigned. MPD, maximum permissible dose. SED, skin erythema dose. ED, an assumed effective depth. BDD, basic dose distribution. wk, week. y, year. P.S., Paris Statement. DEL, dose equivalent limit. [a]"Other organs" refers to organs other than whole body, lens, blood-forming organs, gonads, thyroid, bone, skin, hands and forearms, feet and ankles, head and neck, unless otherwise specified. [b]Or energy equivalent (X-, γ- and β-rays). [c]The doses shown are additional to the natural background radiation. [d]MPD for low penetrating radiation. [e]The first specific advice was provided for pregnant women, but numerical recommendations were not made. [f]A dose equivalent limit (an equivalent dose limit in the modern term). [g]The doses shown are from all sources of internal and external radiation but exclude the dose from medical exposure and the natural background radiation. [h]The value was assigned for individual adults who work in the vicinity of controlled areas or those who enter controlled areas occasionally. [i]The value was assigned for any individuals of the public (i.e., including children and pregnant women) who reside in the neighborhood of controlled areas. [j]The doses shown are from internal and external sources imposed by the occupation and exclude the dose from medical exposure, the natural background radiation and other exposure received by the individual as a member of the public. [k]Internal exposure of single organs. [l]Shown is dose to critical organs. It was also recommended that single doses of the order of 3 rem be avoided as far as practicable, especially in the case of women of reproductive age. [m]For the abdomen of women of reproductive age. [n]When a pregnancy has been diagnosed, the average dose to her fetus should not exceed 1 rem during the remaining period of the pregnancy. [o]The value was assigned for any individual members of the population at large (including persons living in the neighborhood of controlled areas, and children). [p]A dose equivalent limit for the whole body (an effective dose limit in the modern term) was based on detriment, but not that for particular organs. This was renamed "effective dose equivalent limit" by the 1978 Stockholm Statement. [q]A dose equivalent limit (an equivalent dose limit in the modern term) of 500 mSv/year was set for all organs other than the lens. [r]An equivalent dose limit. "hands and forearms" and "feet and ankles" have been changed to "hands and feet" since ICRP-60. So, an equivalent dose limit has not been given to forearms and ankles.

Supplementary Table 9. Key papers that influenced the decision of ICRP on the dose limit for the lens

Papers	Impact on ICRP	Major findings of each paper[a] and our comments[b]
Cogan et al., 1949 [S2]	Recognition of radiation cataract in the 1950 Recommendations.[d]	A preliminary report on A-bomb cataracts. Ten cases of radiation cataract were indentified in the survivors who were all within 950 m of the hypocenter at the time of the bombing. Of these, the failure of vision was observed in 7 cases. *The dose was unknown*.
Abelson and Kruger, 1949 [S3]	Recognition of radiation cataract in the 1950 Recommendations.[d]	A preliminary report on neutron cataracts in cyclotron workers. Ten cases of lens opacities were identified for which estimated total exposure to neutrons ranged from 10-135 n (roughly corresponding to 20-270 rem). Of these, vision was definitely impaired in three cases exposed to 80-135 n. Fast neutrons were considered as the most likely causative agent, though contribution of another cataractogenic radiation of γ-rays and radiofrequency radiation was also considered.
Ham, 1953 [S4]	Definition of a special QF of 30 for high-LET radiation cataract in ICRP-6.	A review of radiation cataract. "It seems probable that the cataractogenic dose of fast neutrons must lie somewhere above 15 rep, and considerably below 45 rep,[c] for men in their 20's and early 30's" *(this was judged from the data in accidentally injured 9 persons with a follow-up period of ≤4 years, among which only one person had cataract)*. "It seems wise to regard 500 r of X- or γ-rays as potentially dangerous to the human lens" *(500 r was chosen from the minimum cataractogenic doses proposed by others, e.g., 500-1000 r (Hunt), ≥1500 r (Merriam) and 600 r (Donaldson). QF of 30 appeared to come from the value obtained when 500 r is divided by 15 rep.*
Cogan and Dreisler, 1953 [S5]	Suggestion of a threshold-type dose response for detectable lens opacities induced by low-LET radiation in ICRP-14.	Analysis of radiation cataract in 40 radiotherapy patients with a follow-up period of ≤13 years. Lens opacities were not found in ≤400 r. The threshold dose for detectable lens opacity appeared to be of the order of 600 r in a single treatment. In three patients, the time of onset of lens changes after exposure became longer with increasing the treatment time.
Britten et al., 1966 [S6]	Suggestion of a threshold-type dose response for detectable lens opacities induced by low-LET radiation in ICRP-14.<br>The scientific basis for a threshold of >8 Sv for vision-impairing cataracts in protracted exposure in ICRP-41.	Analysis of radiation cataract in 104 radiotherapy patients with a follow-up period of ≤12 years. Of these, there were 9 cases of minor opacities, and 5 cases of vision-impairing cataract. "4000 R to the center of the lens appear to lead to cataract in all cases, less than 2000 R to no cataracts".
Merriam and Focht, 1957 [S7]	Suggestion of a threshold-type dose response for detectable lens opacities induced by low-LET radiation in ICRP-14.<br>The scientific basis for 15 rem/year for low-LET radiation, and the belief that the opacities progressively increase in size and denseness or remain stationary in ICRP-14.	Analysis of 97 radiotherapy patients with radiation cataract with a follow-up period of ≤31 years. A radiation cataract was defined as any clinically recognizable opacity having the characteristic appearance, irrespective of whether vision was affected. The minimum cataractogenic dose was 200 r for single treatments, 400 r for multiple or divided treatments from 3 weeks to 3 months, and 550 r for treatmens from 3 months to 9 years where 20, 49 and 28 cases (i.e., a total of 97 cases) were categorized in these groups, respectively. "All cases which received over 1150 r to the lens, irrepective of the duration of treatment, developed cataracts". "The longer the duration of treatment the lower the incidence at a given dosage range below 1150 r". "The higher the dose for a given duration of treatment, the shorter the time of appearance of the lens changes and the higher the incidence of progressive opacities". "Fractionation delays the time of onset of cataracts and results in fewer severe opacities". *ICRP-14 employed 550 r as the scientific basis for 15 rem/year for low-LET (i.e., the threshold dose for vision-impairing cataract), but 550 r shown by Merriam and Focht was the minimum dose to produce detectable lens opacities.* "For treatments from 3 weeks to 3 months, all 20 cases were with progressive cataracts over 1450 r" *(This is wrong, and it should be corrected to "all 12 cases were with progressive cataracts over 2400 r"). It can also be mentioned that for treatments from 3 months to 9 years, all cases were with progressive cataracts over 2300 r*.
Ham, 1960 [S8]	The scientific basis for 15 rem/year for high-LET radiation in ICRP-14.<br>Suggestion to withdraw an additional modifying factor for QF >1 in ICRP-14.	A review on effects of fast neutrons. "It seems likely that 75 rad is a reasonable estimate of the acute dose of fast neutrons which will cause lens damage in humans". "It would seem wise to regard a cumulative dose of 75-100 rad as likely to approach the threshold for cataract in man". *This value was not derived from the evidence in neutron-exposed humans*. *Rather, the generally considered cataractogenic threshold X-ray dose of 400-500 rad was divided by RBE for fast neutrons of 5, yielding 80-100 r. The lower part was changed from 80 r to 75 r according to the data on monkeys*.
Donaldson, 1952 [S9]	The scientific basis for the spontaneous regression/disapperance of minor opacities with time in ICRP-14.[d]	Reexamination of 70 A-bomb survivors with known radiation cataract history in summer of 1951 (i.e., 6 years after the bombing). There was moderate regression of opacities in 2%, slight regression in 17%, no change in 36%, slight progression in 15%, moderate progression in 26%, and marked progression in 4%.
Cogan et al., 1952 [S10]	Characteristic appearance of radiation-induced lens opacities in ICRP-14.[d]	The clinical features of human radiation cataracts are the doughnut-shaped configulation, and sharply demacrated anterior boundary of the opacity and the bivalve configuration of the opacities.
Merriam et al., 1972 [S11]	The scientific basis for a threshold of >8 Sv for vision-impairing cataracts in protracted exposure, 5 Sv for vision-impairing cataracts in single exposure, 5 Sv for detectable opacities in protracted exposure, and 2 Sv for detectable opacities in single exposure in ICRP-41.	A review on radiation effects on the eye. Analysis of radiation cataract in 223 cases (of these 128 with radiation cataracts) with a follow-up period of 35 years. This analysis was based on the data reported in 9 papers including aforementioned Merriam and Focht in 1957. The lowest dose at which a progressive cataract was observed was 5 Gy. A minimum cataractogenic, single dose of photon irradiation may be about 2 Gy, but a dose of about 7.5 Gy may have a probability approaching unity for the production of a cataract (Merriam et al. used cataract to mean "detectable" opacities). *Though ICRP-41 described by citing this paper that the incidence reached 100% after 7.5 Gy in a single exposure, this paper discribed that about 50% of detectable opacities may be progressive with loss of vision*. The minimum cataractogenic dose (i.e., minimum detectable opacity dose) was 2 Gy for a single exposure, 4 Gy for exposures over periods of 3 weeks to 3 months, and 5.5 Gy for exposures over periods greater than 3 months. *A description in ICRP-14 should be corrected to '5.5 Gy when the dose was fractionated over a period greater than 3 months' but not a period of 3 weeks to 3 months*. The analyzed data described in this paper were quoted from a previously published book [S13].
Morita and Kawabe, 1979 [S12]	The scientific basis for a threshold of >8 Sv for vision-impairing cataracts in protracted exposure in ICRP-41.	Analysis of radiation cataract in 28 radiotherapy patients with a follow-up period of 9 years. No visual impairment was seen 3-9 years after radiation therapy when 28 lenses on the unaffected side protected with a tungsten received <14 Gy in 6 weeks. An impaired or completed loss of vision chiefly due to radiation cataract was observed in every cases when more than 27 Gy was irradiated to the lens.

Supplementary Table 9. *Continued*

Papers	Impact on ICRP	Major findings of each paper[a] and our comments[b]
Otake and Schull, 1982 [S14]	The scientific basis for a threshold of 2 Sv for detectable opacities in single exposure in ICRP-41.	Analysis of posterior lenticular opacities in 2125 A-bomb survivors in Hiroshima and Nagasaki subjected to the routine AHS clinical examinations in 1963 or 1964 (i.e., 17 or 18 years after the A-bombing). "A lenticular opacity in this context implies an ophthalmoscopic and slit lamp biomicroscopic defect in the axial posterior aspect of the lens which may or may not interfere measurably with visual acuity. Several dose-response models were fitted to the data under the tentative 1965 (T65) dose estimates, Oak Ridge National Laboratory (ORNL) or Lawrence Livermore National Laboratory (LLNL) estimates. A γ threshold was significant at 1% level when the data were fitted in 'linear γ: linear neutron' model and 'linear-quadratic γ: linear neutron' model for ORNL and LLNL estimates. Such thresholds ranged from 1.09-1.25 (95% CI: 0.58, 1.85). *A description in ICRP-14 shoud be corrected to 'the threshold of low-LET radiation for increasing the frequency of ophthalmologically detectable opacities has been estimated to approximate 0.6-1.9 Gy', instead of 0.6-1.5 Gy. Note that 'linear-linear model without threshold' was significant for T65, suggestive of no threshold.*
Roth et al., in 1976 [S15]	The scientific basis for a threshold of 3-5 Gy of high-LET radiation for vision-impairing cataracts in ICRP-41.	Analysis of the ocular complications in 93 eyes of radiotherapy patients with a follow-up period of 31 months. The incidence of radiation cataract was 4% (n = 1/28) at ≤1 Gy, 4% (1/28) at 1.01-3 Gy, 31% at 3.01-5 Gy (4/13), 0% at 5.01-10 Gy (0/8), and 44% at >10 Gy (7/16). *Considering no catatacts in patients exposed to 5.01-10 Gy, it would be difficult to judge that 3-5 Gy is a threshold.*
Otake and Schull, 1990 [S16]	The scientific basis for a threshold of 0.5-2 Gy of low-LET radiation for detectable lens opacities in ICRP-60.	Analysis of posterior lenticular opacities in 1983 A-bomb survivors in Hiroshima and Nagasaki in the AHS population. Several dose-response models were fitted to the data under the T65 dose estimates revised (T65DR) and the dosimetry system 1986 (DS1986). A γ threshold was significant at 5% level when the data were fitted in 'linear γ: linear neutron' model for T65DR estimates, and such a threshold was 1.35 Gy (95% CI: 0.53, 2.01). In contrast, γ thresholds were insignificant and its lower bound of 95% CI included zero dose, when the data were fitted to other 5 different models. This suggests a non-threshold-type dose response.
NCRP, 1989 [S17]	The scientific basis for a threshold of 2-10 Gy of low-LET radiation for vision-impairing cataracts in ICRP-60.	"The human evidence suggests a single-dose threshold of about 2 Gy" and "doses of greater than 10 Gy and less than 14 Gy spread over weeks are required to induce a 100% incidence of cataracts" for low-LET radiation. *Thus, this report did not conclude a threshold of 2-10 Gy of low-LET radiation for vision-impairing cataracts. On the other hand, a single dose of 2-10 Gy and a fractionated dose of 4 x days$^{0.17}$ were described as estimated radiotherapy thresholds for cataracts by citing the UNSCEAR 1982 report* [S28] *and Merriam et al., 1972. In turn, the UNSCEAR 1982 report decribed these threshold doses by citing Merriam et al, 1972, but Merriam et al., 1972 did not mention such threshold doses. Namely, these thresholds were described in ICRP, NCRP and UNSCEAR documents through mutual citations without the scientific basis.*
Charles and Brown, 1975 [S18]	The scientific basis for the geminative epithelium as cells most at risk in ICRP-89.	Cells in the germinative equatorial lens epithelium are most at risk in the eye from radiation. The minimum depth of the cells at risk is 2.3 ± 0.4 mm, confirming the long established value of 3 mm for the effective depth of the lens.
Shore and Worgul, 1999 [S19]	Suggestions of minor opacities after <2 Gy in ICRP-92.	A review on the epidemiology of radiation cataracts. A dose threshold at >2 Sv, at about 1.5 Sv or no threshold were suggested for radiation-induced opacities. The available data suggest that there is sparing effect at higher total doses, and that the time for cataract induction may be inversely related to the dose to the lens.
Albert et al., 1968 [S20]	Suggestions in ICRP-92 that if there is a threshold, it may be as low as 0.5 Gy.	Analysis of the posterior subcapsular opacities and visual acquity in 306 radiotherpy patients with a median post-treament follow-up period of 10 years. There was a small but significantly higher incidence of posterior subcapsular opacities of which the severity was very mild. There was no difference in the visual acquity of the irradiated and control cases. The estimated dose to the eye was 0.5 Gy. *No information on the dose response and threshold was given*.
Belkacemi et al., 1996 [S21]	Suggestions of sparing effects for lens opacities in ICRP-92.	Analysis of 494 whole body irradiated patients with a median follow-up period of 5 years. The dose rate delivered was catagorized into three groups: LOW (<48 mGy/min, 157 patients), MEDIUM (48-90 mGy/min, 301 patients) and HIGH (>90 mGy/min, 36 patients). The cataract incidence in HIGH and MEDIUM was significantly higher than that in LOW, but the difference between HIGH and MEDIUM was insignificant.
Nakashima et al., 2006 [S22]	The scientific basis for acute dose threshold for vision-impairing cataract in ICRP-118.	Reanalysis of Minamoto et al., in 2004 [S29] cited in ICRP-103. Diagnosed by a single ophthalmologist using LOCS II (previously three ophthalmologists). DS02 eye doses were used (previuosly DS85 eye doses). The *in utero* group was excluded. A dose threshold of 0.6 Gy (90% CI: <0, 1.2) for cortical cataracts and 0.7 Gy (90% CI: <0, 2.8) for posterior subcapsular opacities in 730 A-bomb survivors participated in AHS during 2000-2002. The dose effect decreased significantly with increasing age at exposure.
Neriishi et al., 2007 [S23]	The scientific basis for acute dose threshold for vision-impairing cataract in ICRP-118.	A non-significant dose threshod of 0.1 Gy (95% CI: <0, 0.8) for prevalence of surgically removed cataracts (479 persons among 3761 A-bomb survivors participated in AHS during 2000-2002, i.e., 55-57 years after exposure) with adjustment for age, gender, city and diabetes. The data were consistent with no threshold and incompatible with a threshold of >0.8 Gy (based on DS02 eye doses wighted as frequency of γ-rays plus 10 times the neutron dose).
Shore et al., 2010 [S24]	A further analysis of Neriishi et al., 2007 cited in ICRP-118.	A review on epidemiological studies of radiation cataracts. A subsequent unpublished analysis of Neriishi et al. in 2007. A best dose-threshold estimate of 0 Gy (95% CI: <0, 0.7 or 0.8) for prevalence of surgically removed cataracts (645 persons among 3994 A-bomb survivors in AHS, 2000-2002) adjusted for age, gender, city and history of diabetes. *This paper dealt with the prevalence of cataract surgery, but not its incidence*.
Blakely et al., 2010 [S25]	A further analysis of Neriishi et al., 2007 cited in ICRP-118.	A meeting report of the 2009 RERF workshop. An unpublished analysis indicating a best dose-threshold estimate of 0.4 Gy (95% CI: <0, 0.8) for incidence of surgically removed cataracts (1028 persons among 6066 A-bomb survivors in AHS, 1986-2005) adjusted for age, gender, city and diabetes. *This is not a further analysis of Neriishi et al., in 2007, and did not describe a threshold of 0.45-0.5 Gy.*

Supplementary Table 9. *Continued*

Papers	Impact on ICRP	Major findings of each paper[a] and our comments[b]
Neriishi et al., 2012 [S26]	A further analysis of Blakely et al., 2010 described but not cited in ICRP-118.[d]	The analysis of the same population used in the unpublished analysis in Blakely et al. in 2010. The estimated threshold dose was 0.45 Gy (95% CI: 0.10, 1.05) for ERR model, and 0.50 Gy (95% CI: 0.10, 0.95) for EAR model for incidence of surgically removed cataracts (1028 persons among 6066 A-bomb survivors in AHS, 1986-2005) adjusted for age, gender, city and diabetes. *Though this paper was not cited, description of "a threshold of 0.45-0.50 Sv for EAR and ERR for incidence of cataract surgery" in ICRP-118 should be derived from the findings of this paper. This paper was submitted in September 2011, whereas the TG63 report was approved for publication (as ICRP-118) in October 2011*.
Worgul et al., 2007 [S27]	The scientific basis for the threshold dose for vision-impairing cataract for fractionated/protracted exposure in ICRP-118.	The eyes of a prospective cohort of 8607 Chernobyl liquidators were asseesed for cataract at 12-14 years after exposure, by using the Marriam-Focht scoring system. The estimated dose thresholds (95% CI) were 0.50 Gy (0.17, 0.95) for stage 1-5 cataract, 0.34 Gy (0.19, 0.68) for stage 1 cataract, 0.50 Gy (0.17, 0.69) for stage 1 non-nuclear cataract, 0.34 Gy (0.18, 0.51) for stage 1 superficial cortical cataract, and 0.35 Gy (0.19-0.66) for stage 1 posterior subcapsular cataract. "Stage 1 cataracts particularly in the more advanced presentation have the potential to affect vision." "Stage 2 cataracts are highly likely to cause impairment that, in some individuals, could be very severe, while Stages 3 and later are certain to cause significant visual loss."

For citation information, see the reference list in the supplementary information. QF, quality factor. LET, linear energy transfer. CI, confidence interval. AHS, Adult Health Study. EAR, excess absolute risk. ERR, excess reletive risk. LOCS, Lens Opacity Classification System. RERF, Radiation Effects Research Foundation. A-bomb, atomic bomb. NCRP, National Council on Radiation Protection and Measurements. UNSCEAR, United Nations Scientific Committee on the Effects of Atomic Radiation.
[a]Sentences with double quotes were taken from each paper without modifications. [b]Our comments are shown in italic fonts. [c]rep (röntgen equivalent physical) was proposed by Parker in 1945, used in the United States, and superseded by rad (1 rep is roughly close to 1 rad). [d]These papers were not directly cited in ICRP publications, but we believe that these papers are the basis.

Supplementary Table 10. Key descriptions with reference to the lens in ICRP publications

ICRP recommended or reported in[a]	Key descriptions
1950	Cataract was included in "the effects to be considered".
1954	The lens was designated as a critical organ. The first dose limit for the lens was defined in the form of maximum permissible dose. An assumed effective depth of 3 mm was defined for calculation of the dose to the lens.
1958 (ICRP-1)	Cataract was included in "the long-term somatic injuries". Ophthalmological examinations should be performed in cases of exposure to neutrons and heavy particles.
1959	Eye shields or other suitable shielding may be necessary to keep the dose in the lens within permissible limits.
1964 (ICRP-6)	The lens was excluded from the critical organs, because a greater importance than other tissues was not assumed when only low-LET radiation was concerned. The lens may be specifically sensitive only to high-LET particulate radiation, and a special QF of 30 was hence assigned to high-LET radiation instead of the usual value of 10 for calculation of the dose equivalent to the lens.
1966 (ICRP-8)	The lens is particularly sensitive to neutrons. Numerical values of cataractogenic dose but not a threshold dose were reported, such that opacities have been observed after single neutron doses of 20-25 rad, but not after 200 rad of X- and γ-rays.
1966 (ICRP-9)	Cataract was included in "somatic effects", and listed as one of the most important effects. Instead of a special QF of 30, a modifying factor as a function of QF was defined for calculation of the dose equivalent to the lens. Such a modifying factor equals (2QF+7)/2 for 1≤ QF <10, and equals 3 for QF ≥10.
1969 (ICRP-14)	A confusion between cataract and opacity was pointed out. Dose limits for the lens should be aimed at preventing vision-impairing cataracts but not at preventing detectable lens opacities. There is the good human evidence suggesting a highly sigmoid dose-response relationship with an apparent threshold for induction of detectable lens opacities. The threshold dose of clinically significant lens opacification is ≥550 rad of low-LET radiation for fractionated exposures spread over 3 months or more, or 75 rad of high-LET radiation received on a single occasion. The belief that the lens is unusually sensitive to high-LET radiation was erroneous. For both low- and high-LET radiation, the occupational dose limit for the lens should be 15 rem/year without an additional factor for QF >1. Opacities progressively increase in size and denseness or remain stationary. Detectable minor lens opacities that do not interfere with vision often do not progress in severity, and may regress or disappear spontaneously with the passage of time. Radiation-induced opacities are often sufficiently characteristic to suggest their cause to a skilled observer. Dose limits can be based directly on human experience, and experimental results need to be used circumspectly.
1975 (ICRP-23)	The germinal area of the lens epithelium with its releatively high mitotic index seems to be a principal area of involvement in the production of cataracts by ionizing radiation. Such area locates approximately 3-4 mm below the surface of the adult eye.
1977 (ICRP-26)	A total dose equivalent of 15 Sv would be below the threshold for the production of any lens opacification that would interfere with vision, when the lens is subjected to protracted irradiation over the occupational lifetime (50 years) with low- or high-LET radiation. Namely, 300 mSv/year is produced by 15 Sv/50 years. For radiation protection purposes, the equator of the lens (most susceptible part of the lens to the induction of lens opacities) can be considered to lie 3 mm behind the surface of the eye.
1980 (B.S.)	At a working lifetime dose equivalent of 15 Sv, some opacities might be produced which, while not themlselves detrimental to vision, might develop without further exposure to the point of causing deterioration of vision.
1984 (ICRP-41)	The lens is among the most radiosensitive tissues of the body. At high doses, lens opacities (or cataracts) develop within months, progress rapidly and eventually cloud the lens completely. At lower doses, the opacities may take years to develop, remain microscopic in size, and cause no significant impairment of vision.<br>The pathogenesis of lens opacities involves damage to dividing cells in the anterior lens epithelium. Damaged cells and their breakdown products migrate posteriorly and accumulate beneath the capsule at the posterior pole, leading to posterior displacement of the lens bow. If enough such damaged cells accumulate, they become visible ophthalmologically as a dot-like central posterior subcapsular opacity. At this stage, the radiogenic opacity has little or no effect on vision and is readily distinguishable from a cataract attributable to other causes. Whether the lesion remains stationary or progresses depends on radiation dose and cannot be predicted on the basis of clinical examination alone. If the lesion progresses, it may eventually involve the anterior cortex and nucleus of the lens, in which case it may untimately impair vision severely. In more advanced stages, the opacity is no longer recognizable as a radiation-induced lesion. The lack of vascularity of the lens, and its consequent hypoxia, are consistent with a high RBE of neutrons for cataractogenesis. Threshold dose was defined as the amount of radiation required to cause a particular effect in at least 1-5% of exposed individuals. The threshold dose equivalent of protracted low-level occupational radiation for vision-impairing cataracts is estimated to exceed 8 Sv, although ophthalmologically detectable opacities might result from smaller doses. Exposure of the lens to 150 mSv each year for 50 years would not cause a vision-impairing cataract, although it might give rise to opacities that could be detected ophthalmologically in some exposed individuals. The effective dose equivalent limit will suffice to prevent the production of nonstochastic effects in members of the public.
1985 (ICRP-44)	The lens is a highly radiosensitive structure, and the equatorial portion of the anterior epithelium is the most sensitive part. Damaged cells from this germinative zone migrate posteriorly and form abnormal fibers that become visible as opacities in the posterior subcapsular region of the lens. Following radiation induction of cataract, excision of the lens and replacement with a plastic prosthesis or an extrinsic corrective lens can restore vision as long as the radiation has not damaged the retina. Examples of calculated mean dose of internal scatters to the lens were provided for irradiation of some target volumes (e.g, the neck).
1990 (ICRP-60)	An equivalent dose limit is needed because the lens makes no contribution to the effective dose. The equivalent dose limit for the skin provides protection for the lens against localized exposure to radiation of lowpenetrating power such as β-particles when applied to the skin of face, and the effective dose limit will be more restrictive for external exposure to penetrating radiation over any substantial part of the whole body. The threshold of annual equivalent dose for visual impairment (catatact) given in ICRP-41 as >150 mSv was confirmed, so that the Commission continued to recommend an annual equivalent dose limit for the lens of 150 mSv.
2003 (ICRP-92)	The mechanism of cataract induction involves a lesion in the proliferative cells of the germinative zone that causes abnormal differentiation resulting in the abnormal lens fibers which in the early stages can be detected in the posterior subcapsular region, a distinguishing feature of radiation induction. Small opacities (i.e., small changes of the lens protein that appear as tiny specs without reduction of visual acuity) appear to evolve from damage to individual cells that undergo abnormal differentiation. Small opacifications must thus be considered as a stochastic response, while their accumulation causes the deterministic effect noted at an opacification level where visual impairment begins. There is considerably less confidence than in the past that a threshold of significant magnitude exists. Some findings raise the question of whether lens opacification should be classified as a deterministic effect. If the damage to the lens is cumulative, the current limit of 150 mSv/year appears too high. If the early small opacities progress with time to a size that may reduce visual acuity (as suggested by some experts), it would be wise to re-examine the recommendations for dose limits. TG should examine all the recent data on the lens and make proposals to the Commission on dose limits for low-LET and other radiation qualities.
2007 (ICRP-103)	Threshold dose was defined as dose estimated to result in only 1% incidence of tissue reactions. The scientific basis and the resulting recommended values of threshold dose for detectable opacities and vision-impairing cataract and equivalent dose limit for the lens were unchanged from ICRP-60, whereas the dose unit for thresholds was changed from Sv to Gy. An equivalent dose limit for the lens is currently being reviewed by TG.
2010 (ICRP-116)	For the lens, the significant volume is that in which the cell nuclei are located. The dosimetric assessments of this report support the continued use of the mean absorbed dose for the lens. C1 will continue to evaluate the location of the stem cells associated with cataract induction.
2011 (S.S.)	For the lens of the eye, the threshold in absorbed dose is considered to be 0.5 Gy. For occupational exposure in planned exposure situations, the Commission recommends an equivalent dose limit for the lens of 20 mSv in a year, averaged over defined periods of 5 years, with no single year exceeding 50 mSv. Optimization of protection should be applied in all exposure situations and for all categories of exposure, and protection should be optimized not only for whole body exposures but also for exposures to specific tissues, particularly the lens.

Supplementary Table 10. *Continued.*

ICRP recommended or reported in[a]	Key descriptions
2012 (ICRP-118)	The newly recommended equivalent dose limit for occupational exposure of the lens is based on prevention of radiogenic cataracts, with the underlying assumption of a nominal threshold at 0.5 Gy for acute or protracted exposure. Nonetheless, optimization is explicitly recommended to aid in keeping doses below the nominal threshold, as well as to account for uncertainties in applying a nominal threshold to a population, and uncertainties in the value and even existence of a threshold.[b]<br>No new limit has been recommended for public exposures of the lens, as the Commission judged that the existing limit was adequately protective, and therefore reduction of the limit could impose unnecessary restrictions. It seems highly improbable that any member of the public would receive a dose to the lens over a lifetime in excess of the nominal threshold of 0.5 Gy in a planned exposure situation considering: application of the effective dose limit of 1 mSv/year; the low likelihood of the lens of the eye being preferentially exposed for any significant period; and optimization of protection below the equivalent dose limit for the lens.[b]<br>A 'true' threshold dose for a given effect can be defined as a dose below which the effect does not occur, but is often difficult to determine. For protection purposes, the ‘practical' threshold dose is defined as the estimated dose for 1% incidence, denoting the amount of radiation required to cause a specific, observable effect in only 1% of individuals exposed to radiation. The use of a smaller level than 1% would entail a greater extrapolation of response frequencies to even lower doses, with concomitant greater uncertainties attached to the value. The use of a higher level would have less uncertainties in the value and may be acceptable in practical situations for some endpoints but not others.<br>Radiation cataract is still considered a tissue reaction with a dose threshold, albeit small. There is no direct evidence that a single damaged progenitor lens epithelial cell can produce a cataract (the hallmark of a stochastic effect with zero threshold), even though the lower 95% CI includes zero dose in some threshold calculations. The precise mechanism of radiation cataractogenesis is not known, but genomic damage resulting in altered transcription, cell division and/or abnormal lens fiber cell differentiation is considered to be the salient injury, rather than cell killing. One theory is that aberrantly dividing and/or differentiating cells in the pre-equatorial region of the lens epithelium migrate, predominately to the lens posterior pole where they become opaque lens fibers. Radiation damage to single lens epithelial or fiber cells probably results in localized small changes in lens transparency. Accumulation and coalescence of these micro-opacities have been suggested to result in populations of damaged lens fiber cells that form larger lens defects, eventually resulting in clinical opacity. Radiation cataract formation has also been suggested to be likely to depend on survival and potential division and/or differentiation of lens epithelial cells with compromised genomes. There is evidence for the importance of cell division and proliferation in cataract formation. It has been shown that the lens epithelium of patients with cataracts harbors an increased frequency of micronuclei (a marker of impaired cell division), and that radiation cataracts will not form in animals if epithelial cell division is totally inhibited or if the dividing epithelial cells are shielded from radiation exposure. Thus, radiation-induced unrepaired DNA damage in such dividing and differentiating lens epithelial cells may be the crucial first step in cataractogenesis, and it can be speculated that radiation cataract formation could be explained by initial damage to a single progenitor epithelial cell in the lens which, upon cell division and differentiation, results in groups of defective lens fiber cells. A genetic component to the radiosensitivity of cataractogenesis has also been suggested which may produce more cataracts in a few percent of exposed individuals: lenses containing cells with impaired ability to recognize and repair such damage are probably at increased risk for cataractogenesis, and heterozygosity for genes involved in cell-cycle checkpoint control, DNA damage recognition or DNA repair might also contribute to this phenomenon. Future research may elucidate the true mechanism of cataract formation.<br>In view of various problems in early studies and reports in the past few years of markedly lower threshold doses deduced from various radiation exposure scenarios, it is prudent to recommend changes to the threshold doses.<br>Acute dose threshold should be lowered to a nominal value of 0.5 Gy. Formal estimates of acute dose threshold were made in two AHS studies of A-bomb survivors that provided threshold doses of 0.1–0.7 Gy with 90–95% CI including 0 Gy. One was Nakashima et al. in 2006 that indicated a threshold of 0.50–0.70 Sv for early opacities. The other was Neriishi et al. in 2007 that indicated a threshold of 0.10 Sv for prevalence of cataract removal surgery. The threshold for fractionated and protracted exposures is not larger than that for acute exposures (i.e., < 0.5 Gy). This estimate was calculated from the data of Worgul et al. in 2007 for stage 1 (early) opacities in the Chernobyl liquidators, and ranged between 0.34 and 0.50 Gy (95% CI: 0.17, 0.69). There was no dependence of threshold dose on stage or site of the cataract. The lack of a sparing effect of dose fractionation for reactions manifesting very late after low total doses (particularly for cataracts) implies that the injury is caused by single-hit irreparable-type events.<br>The threshold for chronic exposure over several to many years is the same as that for acute exposure (i.e., 0.5 Gy for both scenarios). The threshold is difficult to judge from the scientific evidence because of the uncertainties about progression of opacities into cataracts and the age-at-exposure problem. The threshold dose values for chronic exposure depend on the exposure duration and the follow-up period after exposure, and differences between these time variables among different studies make the values more uncertain. The same incidence of injury is assumed irrespective of the acute or chronic nature of the exposure over a working life, with >20 years follow-up.<br>The threshold dose for radiation cataract is considered to be around 0.5 Gy (i.e., the same threshold assumed for acute, fractionated/protracted and chronic exposures in the absence of evidence).<br>The public annual threshold dose values would be scaled down in proportion to relative lifespan minus latency period of 20 years for the lens vs working life, though great uncertainty is attached to these values.<br>Acute doses up to around 100 mGy produce no functional impairment of tissues including the lens, with the caveat that the use of a threshold model remains uncertain for the lens. The stochastic risks of cancer and hereditary effects remain the principal risks to consider for most applications of ICRP recommendations in occupational or public situations. The risk of tissue reactions (particularly for the lens) becomes increasingly important, at very long times after exposure to acute or accumulated doses of >0.5 Gy (particularly regarding radiation incidents and accidents and medical exposures).

LET, linear energy transfer. QF, quality factor. B.S., Brighton Statement. TG, Task Group. C1, ICRP Committee 1. S.S., Seoul Statement. CI, confidence interval. AHS, Adult Health Study. A-bomb, atomic bomb. [a]Reports are shown in italic fonts. [b]Described in the Guest Editorial of ICRP-118.

Supplementary Table 11. Changes in dose limit for special, short-term, abnormal, accidental or emergency exposures

ICRP recommended or reported in[n]	Exposure situations	Exposure types	Subject	Dose limitation or restriction
1958 (ICRP-1)	Accidental exposure[a]	External/internal	Workers	25 rem/accident (whole body)[b]
	Emergency exposure[c]	External/internal	Workers	12 rem (whole body)[d]
1959	Accidental exposure[a]	External/internal	Workers	25 rem/accident (whole body)[b]
	Emergency exposure[c]	External/internal	Workers	12 rem (whole body)[d]
	Emergency exposure[e]	Internal	Workers	12 rem (whole body, gonads), 30 rem (skin, thyroid, bone), 15 rem (other organs excluding lens)
	Short-term exposure[e]	Internal	Workers	3 rem (whole body, gonads), 8 rem (skin, thyroid, bone), 4 rem (other organs excluding lens)
1964 (ICRP-6)	Accidental exposure[a]	External/internal	Workers	25 rem/accident (whole body)[b]
	Emergency exposure[c]	External/internal	Workers	12 rem (whole body)[d]
	Emergency exposure[e]	Internal	Workers	5 rem (whole body, gonads, blood-forming organs), 30 rem (skin, thyroid, bone), 15 rem (other organs excluding lens)
	Short-term exposure[e]	Internal	Workers	1.3 rem (whole body, gonads, blood-forming organs), 8 rem (skin, thyroid, bone), 4 rem (other organs excluding lens)
1966 (ICRP-9)	Emergency exposure	External/internal	Workers	It is impossible to specify dose limits.
	Accidental exposure	External/internal	Workers	It is impossible to specify dose limits. The worker should be subject to health supervision if twice the annual dose limit is exceeded.
	Planned special exposure[c,f]	External/internal	Workers	Twice the annual dose limit for critical organs in any single event, 5 times the annual dose limit for critical organs in a lifetime.
	Short-term exposure	Internal	Workers	1/2 of the annual dose limit in 1/4 of a year
1978 (ICRP-28)	Abnormal exposure	External	Workers	Essentially administrative action: greater than the relevant annual dose-equivalent limit but not exceeding twice the annual dose-equivalent limit. More detailed administrative enquiry with an assessment of possible biological consequences: exceeding twice the annual dose-equivalent limit but not 5 times the annual dose-equivalent limit. Above actions supplemented by an examination of the exposed worker by the physician: exceeding 5 times the annual dose-equivalent limit.
		Internal	Workers	Making the best possible evaluation of the intake and considering therapy: greater than the annual limit of intake.
1990 (ICRP-60)	Emergency exposure[g]	External/internal	Workers	Effective dose of about 0.5 Sv and equivalent dose to the skin of about 5 Sv except for life saving. No dose restrictions for life saving by informed volunteers (not described in ICRP-60 but described in ICRP-103).
1992 (ICRP-63)	Emergency exposure[h]	External/internal	Public	Sheltering: 50 mSv (effective dose). Administration of stable iodine: 500 mSv (equivalent dose to the thyroid). Evacuation (<1 week): 500 mSv (effective dose), 5 Sv (equivalent dose to the skin). Relocation: 1 Sv (effective dose).
		Internal	Public	Restriction to a single foodstuff: 10 mSv/year.
2005 (ICRP-96)	Emergency exposure[i]	External/internal	Workers[j]	Rescue operations (saving life): no dose restrictions (if the benefit to others clearly outweighs the rescuer's own risk). Rescue operations (preventing serious injury or the development of catastrophic conditions): every effort should be made to avoid deterministic effects on health - by keeping effective dose below 1000 mSv to avoid serious deterministic health effects, or below 10 times the maximum single year dose limit to avoid other deterministic health effects (e.g., effective dose of 500 mSv, equivalent dose to the lens of 1500 mSv). Other rescue operations (immediate and urgent actions to prevent injuries for large doses to many people): below twice the maximum single year limits (e.g., effective dose of 100 mSv, equivalent dose to the lens of 300 mSv). Other operations (e.g., recovery and restoration operations): normal occupational dose limits (e.g., equivalent dose to the lens of 150 mSv/year).
	Emergency exposure[k]	External/internal	Public	Sheltering: effective dose of ~10 mSv in 2 days. Temporary evacuation: effective dose of ~50 mSv in 1 week. Relocation: effective dose of ~1000 mSv or ~100 mSv first year. Iodine prophylaxis (if radioiodines are involved): equivalent dose to the thyroid of ~100 mSv.
2007 (ICRP-103)	Emergency exposure[l]	External/internal	Workers	The same as ICRP-96 but in reference levels.[m]
		External/internal	Public	In planning, typically between 20 and 100 mSv/year according to the situation.
	Existing exposure[l]	External/internal	Public	Between 1 and 20 mSv/year according to the situation.

[a]Defined as exposure that occurs only once in a lifetime. [b]Dose higher than 25 rem was considered potentially serious. [c]Women of reproductive age or capacity shall not be subjected to these exposures. [d]The accumulated dose should conform with 5 rem/year within a period not exceeding 5 years. [e]The 50 year integrated dose. [f]Such exposures should not be permitted if the addition of intended dose to the worker's accumulated dose exceeds 5(N-18) or 5 rem/year (see Supplementary Table 7), or if the worker has received, in the previous 12 months, a single exposure or intake of radioactive materials with a dose commitment in excess of the quarterly quota, or if the worker has previously received abnormal exposures (i.e., accidental or emergency exposures) in excess of 5 times the annual dose limit. [g]Intervention level (the level of avertable dose at which a specific protective action or remedial action is taken in an emergency exposure situation or chronic exposure situation). [h]The almost always justified intervention level of averted dose. [i]Dose guidance values for restraining the occupational exposure of responders. [j]Under conditions that may lead to doses above normal occupational exposure limits, workers should be volunteers and should be instructed in dealing with radiation hazards to allow them to make informed decisions. Female workers who may be pregnant or are nursing an infant should not be employed as first responders undertaking life saving or other urgent actions. [k]Avertable dose for which the countermeasure is generically optimized. [l]Reference level (in emergency or existing controllable exposure situations, this represents the level of dose or risk, above which it is judged to be inappropriate to plan to allow exposures to occur, and below which optimization of protection should be implemented. The chosen value for a reference level will depend upon the prevailing circumstances of the exposure under consideration. Not applicable to existing occupational exposure). [m]Long-term recovery operations, and exposures resulting from long-term remediation operations or from protracted employment in affected areas, should be treated as part of planned occupaitonal exposure. [n]Reports are shown in italic fonts.
